# Prospects for ${K_L \rightarrow \pi ^0\nu \bar{\nu }}$, $K_S\rightarrow \mu ^+\mu ^-$, $K_L\rightarrow \pi ^0 \ell ^+\ell ^-$ and $\varepsilon '/\varepsilon$ after the new ${K^+ \rightarrow \pi ^+\nu \bar{\nu }}$ result from NA62

**DOI:** 10.1140/epjc/s10052-026-16418-0

**Published:** 2026-10-13

**Authors:** Monika Blanke, Andrzej J. Buras, Cristina Lazzeroni, Joel C. Swallow

**Affiliations:** 1 Institut für Astroteilchenphysik, Karlsruhe Institute of Technology, Hermann-von-Helmholtz-Platz 1, 76344 Eggenstein-Leopoldshafen, Germany; 2 Institut für Theoretische Teilchenphysik, Karlsruhe Institute of Technology, Engesserstraße 7, 76128 Karlsruhe, Germany; 3 TUM Institute for Advanced Study, Lichtenbergstr. 2a, 85748 Garching, Germany; 4 Physik Department, TUM School of Natural Sciences, TU München, James-Franck-Straße, 85748 Garching, Germany; 5 University of Birmingham, Edgbaston, Birmingham, B15 2TT UK; 6 CERN, Esplanade des Particules 1, 1211 Meyrin, Switzerland

## Abstract

The recently announced NA62 measurement of the ${K^+ \rightarrow \pi ^+\nu \bar{\nu }}$ branching ratio, based on 2016–2024 data, is fully consistent with its very accurate Standard Model (SM) prediction. While it is not excluded that the final result based on 2016–2026 data will deviate from the SM prediction, the question arises whether a similar fate awaits the ${K_L \rightarrow \pi ^0\nu \bar{\nu }}$ decay. This decay is currently being searched for by the KOTO experiment, with the present upper bound roughly two orders of magnitude above its very precise SM prediction. The proposed KOTO II experiment aims to provide the first discovery of the ${K_L \rightarrow \pi ^0\nu \bar{\nu }}$ decay and measure its branching ratio. Building on the findings of several previous papers we demonstrate that in the presence of suitably chosen large new complex phases and of a small amount of new right-handed $\bar{s} d$ couplings, in addition to the left-handed ones, the ${K_L \rightarrow \pi ^0\nu \bar{\nu }}$ branching ratio can still be enhanced by one order of magnitude with respect to the SM prediction while keeping both ${K^+ \rightarrow \pi ^+\nu \bar{\nu }}$ and $\varepsilon _K$ SM-like. Simultaneously the decays $K_S\rightarrow \mu ^+\mu ^-$, studied by LHCb, and $K_L\rightarrow \pi ^0\ell ^+\ell ^-$, searched for by KOTO II, can be strongly enhanced and the anomaly in the ratio $\varepsilon '/\varepsilon$, as claimed by Dual QCD, removed. We illustrate this with an example of a specific $Z^\prime$ scenario.

## Introduction

Rare decays of kaons have, for decades, played a very important role in testing the Standard Model (SM) and in the search for new physics (NP). Recent reviews of these decays can be found in [1, 2]. Table 2 in [1] demonstrates in an impressive manner the interest in these decays in the context of the search for NP.

The main players in this game are the decays

1 $$\begin{aligned}  &   {K^+ \rightarrow \pi ^+\nu \bar{\nu }},\quad {K_L \rightarrow \pi ^0\nu \bar{\nu }},\quad K_L\rightarrow \mu ^+\mu ^-, \nonumber \\  &   \quad K_S\rightarrow \mu ^+\mu ^-,\quad K_L\rightarrow \pi ^o\ell ^+\ell ^-(\ell = e,\mu ), \end{aligned}$$

as well as the two CP-violating observables

2 $$\begin{aligned} \varepsilon _K, \quad \varepsilon '/\varepsilon . \end{aligned}$$

The status of these six rare decays and of the two observables, summarised recently in [3], is as follows:

- The NA62 collaboration at CERN recently published a measurement of the ${K^+ \rightarrow \pi ^+\nu \bar{\nu }}$ branching ratio based on 2016–2024 data with the result [4]^1^
  3 $$\begin{aligned} \mathcal {B}({K^+ \rightarrow \pi ^+\nu \bar{\nu }})_\text {exp}=(9.6^{+1.9}_{-1.8})\times 10^{-11}\,. \end{aligned}$$
  This is fully consistent with the SM estimates given below in (15) and (17) but still leaving room for some NP contributions. The NA62 experiment will complete collecting its full dataset in 2026, with the 2025–2026 data—amounting to approximately 1/3 of the full 2016–2026 dataset—still to be analysed.
- The most recent 90% confidence level (C.L.) upper bound on ${K_L \rightarrow \pi ^0\nu \bar{\nu }}$ from KOTO [6] reads
  4 $$\begin{aligned} \mathcal {B}({K_L \rightarrow \pi ^0\nu \bar{\nu }})_\text {exp}\le 2.2\times 10^{-9}\,, \end{aligned}$$
  roughly two orders of magnitude above the SM predictions given in (15) and (17). The KOTO experiment will improve its sensitivity by an order of magnitude by 2030, and “an indication of new physics with a significance of 90% is possible if the observed branching ratio differs by 40% from the SM value” [7]. The proposed KOTO II experiment [8] targets the first discovery of the ${K_L \rightarrow \pi ^0\nu \bar{\nu }}$ decay and a measurement of its branching ratio in the late 2030s.
- The decay $K_L\rightarrow \mu ^+\mu ^-$ is subject to significant long-distance contributions. Nonetheless an experimental bound on the short-distance (SD) contribution, sensitive to NP, can be derived [9]:
  5 $$\begin{aligned} \mathcal {B}(K_L\rightarrow \mu ^+\mu ^-)_\textrm{SD} \le 2.5 \times 10^{-9}\,. \end{aligned}$$
- The present upper bound on $K_S\rightarrow \mu ^+\mu ^-$ from the LHCb collaboration, using data from 2011–2012 and 2016–2018 (Run1 + Run2), reads [10]
  6 $$\begin{aligned} \mathcal{B}(K_S\rightarrow \mu ^+\mu ^-)_\textrm{LHCb}&< 2.1 \times 10^{-10} \; \text{ at } \; 90\% \; \text{ C.L. } \,. \end{aligned}$$
  This result is about a factor of 50 above the SM prediction given in (39) [9, 11]. LHCb expects to significantly improve the sensitivity using Run3 data, with strong prospects to make a measurement of the branching ratio by the end of LHC operations [12].
- The present experimental bounds on the decays $K_L\rightarrow \pi ^o\ell ^+\ell ^-$ from the KTeV experiment are
  7 $$\begin{aligned}  &   \mathcal{B}(K_L\rightarrow \pi ^0e^+e^-)<28\times 10^{-11}\quad [13],\nonumber \\  &   \mathcal{B}(K_L\rightarrow \pi ^0\mu ^+\mu ^-)<38\times 10^{-11}\quad [14], \end{aligned}$$
  which are still by one order of magnitude larger than the respective SM predictions. The KOTO II proposal includes plans to measure the branching ratios of these decays [8].
- The $K^0-\bar{K}^0$ CP-violating parameter $\varepsilon _{K}$ is fully consistent with the SM calculations, as emphasised in [15, 16].^2^
- The ratio $\varepsilon '/\varepsilon$ quantifying the amount of direct CP violation in $K_L\rightarrow \pi \pi$ decays, as measured by the NA48 collaboration at CERN [17] and KTeV at Fermilab [18, 19], is at least a factor of two above the SM prediction obtained using the Dual QCD approach [20]. While the results from the RBC-UKQCD collaboration [21] using lattice QCD methods and from Chiral Perturbation Theory [22, 23] are consistent with the experimental value, large uncertainties do not allow definite conclusions to be drawn. Recent reviews on this topic can be found in [1, 24].

As evident from many papers listed in Table 2 of [1] and in Table 8 of [25], the observables listed in (1) and (2) can, in certain NP scenarios, be strongly correlated with each other. A recent SMEFT analysis in the context of a particular $Z^\prime$ model presented in [26] demonstrated this in an impressive manner: assuming the flavour-changing $\bar{s} d Z^\prime$ coupling to be purely left-handed (LH) and imaginary, a possible enhancement of the ${K^+ \rightarrow \pi ^+\nu \bar{\nu }}$ branching ratio implies enhancements of the rare decays ${K_L \rightarrow \pi ^0\nu \bar{\nu }}$, $K_S\rightarrow \mu ^+\mu ^-$, $K_L\rightarrow \pi ^{0}\ell ^+\ell ^-$ and of $\varepsilon '/\varepsilon$ in a correlated manner while keeping $\varepsilon _K$ fully consistent with the SM expectations and its experimental value, as seen in Figs. 2 and 3 of [26].

**Fig. 1:**
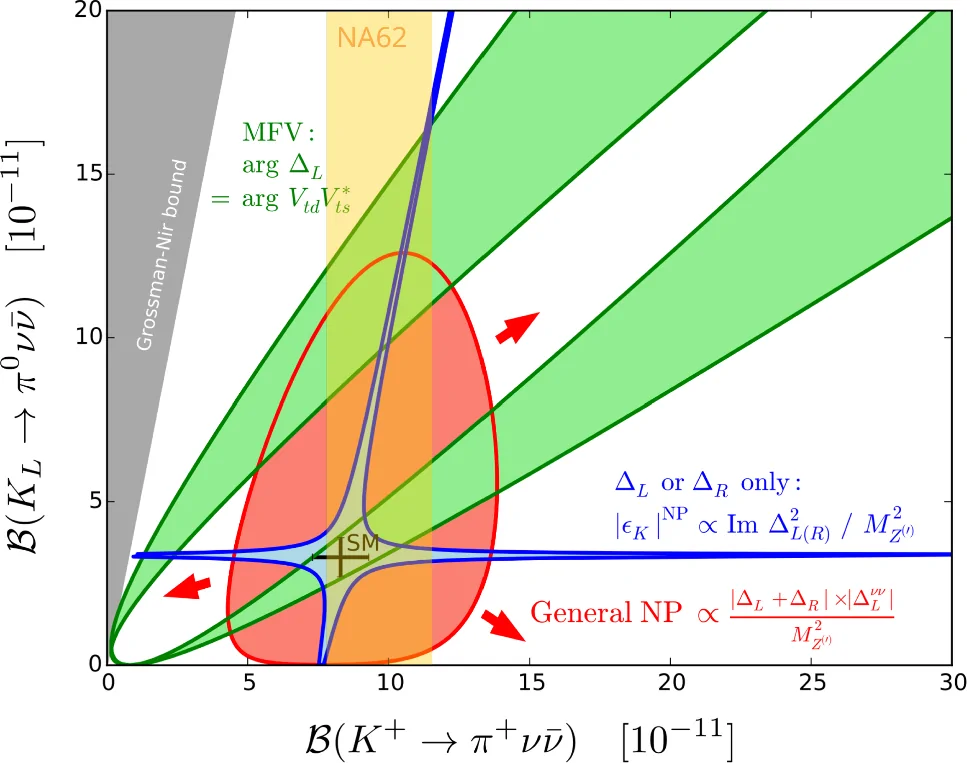
Illustrations of common correlations in the $\mathcal {B}({K^+ \rightarrow \pi ^+\nu \bar{\nu }})$ versus $\mathcal {B}({K_L \rightarrow \pi ^0\nu \bar{\nu }})$ plane. The expanding red region illustrates the lack of correlation for models with general LH and RH NP couplings. The green region shows the correlation present in models obeying Constrained Minimal Flavour Violation [32–34]. The blue region shows the correlation induced by the constraint from $\varepsilon _K$ if only LH or RH couplings are present [31]. The yellow band shows the values compatible with the NA62 measurement within 1σ [4]. Adapted from [27]

As seen in Fig. 1, adapted from [27] to include the new NA62 measurement, looking at $\mathcal {B}({K_L \rightarrow \pi ^0\nu \bar{\nu }})$ vs $\mathcal {B}({K^+ \rightarrow \pi ^+\nu \bar{\nu }})$, the enhancements found in [26] take place in the blue branch parallel to the Grossman-Nir bound [28]. The type of correlation between $\mathcal {B}({K_L \rightarrow \pi ^0\nu \bar{\nu }})$ and $\mathcal {B}({K^+ \rightarrow \pi ^+\nu \bar{\nu }})$ implied by the $\varepsilon _K$ constraint in the presence of only LH or right-handed (RH) couplings had already been found in the context of the Littlest Higgs model with T-parity [29] and in the minimal 3-3-1 model [30]. The general insight into such a correlation has been provided in [31], and subsequently illustrated in an analysis of simplified models with LH and RH couplings [27].

It is evident from Fig. 1 that the new NA62 measurement, together with the absence of NP in $\varepsilon _K$, implies a possible enhancement of the ${K_L \rightarrow \pi ^0\nu \bar{\nu }}$ rate by at most a factor of five in models with only LH or RH couplings, well below the model-independent Grossman-Nir bound [28]. If eventually ${K^+ \rightarrow \pi ^+\nu \bar{\nu }}$ were proven to be essentially unaffected by NP, any significant enhancements of $\mathcal {B}({K_L \rightarrow \pi ^0\nu \bar{\nu }})$, $\mathcal {B}(K_S\rightarrow \mu ^+\mu ^-)$ and $\varepsilon '/\varepsilon$, as found in [26], would be excluded in the case of only LH or RH couplings. The presence of both LH and RH couplings is therefore required in order to populate regions outside the two blue branches.

In our view, the current experimental situation does not warrant a detailed sophisticated SMEFT analysis of the type presented in [26]. Nevertheless, this will be of significant interest in the future, after the final result from the NA62 collaboration is published, the bounds for $\mathcal {B}({K_L \rightarrow \pi ^0\nu \bar{\nu }})$, $\mathcal {B}(K_S\rightarrow \mu ^+\mu ^-)$ improved and the situation of $\varepsilon '/\varepsilon$ within the SM clarified. The goal of our paper is, instead, to demonstrate in simple terms that indeed in the presence of both LH and RH $\bar{s} d$ couplings with new CP-violating phases ${K_L \rightarrow \pi ^0\nu \bar{\nu }}$, $K_S\rightarrow \mu ^+\mu ^-$, $K_L\rightarrow \pi ^o\ell ^+\ell ^-$ and $\varepsilon '/\varepsilon$ can be significantly enhanced while keeping both ${K^+ \rightarrow \pi ^+\nu \bar{\nu }}$ and $\varepsilon _K$ SM-like.

Our paper is organised as follows. In Sect. 2 we collect the most important formulae used in our paper. In Sect. 3 a specific $Z^\prime$ scenario is presented, for which the numerical analysis is performed in Sect. 4. We end our paper with a short summary and an outlook for the coming years.

## General formulae

In this section we provide the general formulae for the branching ratios of the relevant kaon decays, both within and beyond the SM, where we allow for the presence of LH and RH quark currents. These expressions are sufficient to get an idea of the structure of various effects and to study them numerically. Further details, in particular the derivations of these formulae, can be found in [35] and in the original papers listed there and below.

### ${K^+ \rightarrow \pi ^+\nu \bar{\nu }}$ and ${K_L \rightarrow \pi ^0\nu \bar{\nu }}$

#### Basic Expressions

The branching ratios of the theoretically extremely clean $K\rightarrow \pi \nu \bar{\nu }$ decays can conveniently be parametrised in terms of a complex function $X_\text {eff}$ containing both SM and NP contributions. Including isospin breaking corrections, summing over the three SM neutrino flavours,^3^ and generalising the SM formulae in [36–38] to include NP effects from both LH and RH quark currents, one finds [27]

8 $$\begin{aligned}  &   \mathcal {B}(K^+\rightarrow \pi ^+\nu \bar{\nu })= \kappa _+ (1+\Delta _\text {EM})\cdot \nonumber \\  &   \left[ \left( \frac{\textrm{Im}X_\textrm{eff}}{\lambda ^5}\right) ^2+ \left( \frac{\textrm{Re}\lambda _c}{\lambda }P_c(X)+ \frac{\textrm{Re}X_\textrm{eff}}{\lambda ^5}\right) ^2\right] , \end{aligned}$$

9 $$\begin{aligned}  &   \mathcal {B}({K_L \rightarrow \pi ^0\nu \bar{\nu }})=\kappa _L\cdot \left( \frac{\textrm{Im}X_\textrm{eff}}{\lambda ^5}\right) ^2, \end{aligned}$$

where $\lambda =|V_{us}|$ and $\lambda _c=V^*_{cs}V_{cd}$. Furthermore $\Delta _\text {EM}=-0.003$ [39] parametrises the electromagnetic corrections from photon exchanges, and the normalisation factors read [39]

10 $$\begin{aligned}  &   \kappa _+={ (5.173\pm 0.025 )\cdot 10^{-11}\left[ \frac{\lambda }{0.225}\right] ^8}, \nonumber \\  &   \kappa _L=(2.231\pm 0.013)\cdot 10^{-10}\left[ \frac{\lambda }{0.225}\right] ^8. \end{aligned}$$

$\kappa _+$ and $\kappa _L$ summarise all factors beyond CKM parameters, in particular the relevant hadronic matrix elements that can be extracted from leading semi-leptonic decays of $K^+$, $K_L$ and $K_S$ mesons [39]. In obtaining the numerical values in (10)

11 $$\begin{aligned} \sin ^2\vartheta _W\equiv s_W^2=0.23116, \qquad \alpha (M_Z)=\frac{1}{127.9}, \end{aligned}$$

given in the $\overline{\text {MS}}$ scheme, have been used. Their errors are below 0.1% and can be neglected. Further details can be found in Section 9.5.3 of [35].

Finally, the function $X_\text {eff}$ can be decomposed as follows:

12 $$\begin{aligned} X_\textrm{eff}=\lambda _t\,(X_L(K)+X_R(K))\equiv \lambda _t X_\textrm{tot} \end{aligned}$$

where $$\lambda _t = V_{ts}^*V_{td}$$, and

13 $$\begin{aligned} X_L(K)=X(x_t)+\Delta X_L(K) \end{aligned}$$

represents contributions of LH currents, where $X(x_t)$ is the SM contribution with $x_t=m^2_t/M^2_W$ and $\Delta X_L(K)$ denotes NP contributions. Likewise $X_R(K)$ represents the contributions of RH currents that are absent in the SM. Note that only the vector parts of these currents contribute to the $K\rightarrow \pi \nu \bar{\nu }$ decays.

The NLO QCD corrections to $X(x_t)$ have been calculated in [38, 40, 41], the NLO electroweak ones in [42] and the NNLO QCD ones recently as stated below. NLO QCD corrections to $P_c(X)$ have been calculated in [43] and the NNLO ones in [44, 45], while the electroweak ones have been obtained in [46]. Finally the long-distance contributions to $P_c(X)$ have been determined in [47].

The 2026 values of $X(x_t)$ and $P_c(X)$ are then given by

14 $$\begin{aligned} X(x_t)=1.475\pm 0.008, \qquad P_c(X)=0.405\pm 0.024\,, \end{aligned}$$

with the latter calculated for λ=0.225. The NNLO value of $X(x_t)$ given above has been extracted in [25] from [48]. It replaces the value $X(x_t)=1.462\pm 0.017$ presented in [49].

Due to the absence of $P_c(X)$ in (9), $\mathcal {B}(K_L\rightarrow \pi ^0\nu \bar{\nu })$ has essentially no theoretical uncertainties. It is only affected by parametric uncertainties coming from $m_t$, $\text {Im}\lambda _t$ and $\kappa _L$, out of which only the one due to $\text {Im}\lambda _t$ is important.

Using the BV strategy of [15, 16], that determines the CKM parameters from ΔF=2 observables and eliminates through ratios the dependence on $|V_{cb}|$, the updated SM predictions of the ${K^+ \rightarrow \pi ^+\nu \bar{\nu }}$ and ${K_L \rightarrow \pi ^0\nu \bar{\nu }}$ branching ratios determined independently of $|V_{cb}|$ and γ read in 2026 [25]

15 $$\begin{aligned} \mathcal {B}({K^+ \rightarrow \pi ^+\nu \bar{\nu }})_\text {SM}= &   (8.65\pm 0.42)\times 10^{-11},\end{aligned}$$

16 $$\begin{aligned} \mathcal {B}({K_L \rightarrow \pi ^0\nu \bar{\nu }})_\text {SM}= &   (3.05\pm 0.17)\times 10^{-11}\, \end{aligned}$$

with the uncertainties in β, $\hat{B}_K$ and $P_c(X)$ taken into account. These are the most precise determinations to date.

The numbers in (15), (16) should be compared with the results of [48] where instead of eliminating $|V_{cb}|$ a global UT fit of CKM parameters has been used:

17 $$\begin{aligned} \mathcal {B}({K^+ \rightarrow \pi ^+\nu \bar{\nu }})_\text {SM}= &   (8.55\pm 0.47)\times 10^{-11} ,\end{aligned}$$

18 $$\begin{aligned} \mathcal {B}({K_L \rightarrow \pi ^0\nu \bar{\nu }})_\text {SM}= &   (3.15\pm 0.23)\times 10^{-11}. \end{aligned}$$

One observes that there is a good agreement between these two ways of obtaining predictions for the $K\rightarrow \pi \nu \bar{\nu }$ branching ratios. This is not surprising since the global CKM fit is mostly based on ΔF=2 observables. Needless to say, the difference between the result in (15) and (17) is, from the point of view of the search for NP in ${K^+ \rightarrow \pi ^+\nu \bar{\nu }}$, immaterial as it is far beyond the precision of the present experimental data and the ones expected in the near future. The significantly larger error in the ${K_L \rightarrow \pi ^0\nu \bar{\nu }}$ branching ratio in (18) is related to the $|V_{cb}|^4$ dependence present in $\mathcal {B}({K_L \rightarrow \pi ^0\nu \bar{\nu }})$ that is absent in the BV strategy.

The latest NA62 measurement of the ${K^+ \rightarrow \pi ^+\nu \bar{\nu }}$ branching ratio, quoted in (3), is fully consistent with the SM estimates, while still leaving room for some NP contributions. For the ${K_L \rightarrow \pi ^0\nu \bar{\nu }}$ mode, the experimental sensitivity has not yet reached the level of the SM prediction, with an upper limit from the KOTO experiment quoted in (4) leaving plenty of room for NP.

#### Interplay of ${K^+ \rightarrow \pi ^+\nu \bar{\nu }}$ and ${K_L \rightarrow \pi ^0\nu \bar{\nu }}$

Once $\mathcal {B}({K^+ \rightarrow \pi ^+\nu \bar{\nu }})$ and $\mathcal {B}({K_L \rightarrow \pi ^0\nu \bar{\nu }})$ have been measured, the complex function $X_\textrm{eff}$ can be determined, subject to ambiguities that we will discuss below.

To this end it is useful to define

19 $$\begin{aligned} X_\textrm{eff}{=}-|X_\textrm{eff}| e^{-i\beta _X}, \quad X_\textrm{tot}{=}|X_\textrm{tot}|e^{i\theta _X}, \quad \beta _X{=}\beta -\beta _s-\theta _X\,, \end{aligned}$$

where

20 $$\begin{aligned} V_{ts}= - |V_{ts}|e^{-i\beta _s}, \qquad V_{td}=|V_{td}|e^{-i\beta }\,. \end{aligned}$$

The values of CKM parameters used in our analysis are collected in Appendix A.

As

21 $$\begin{aligned} \textrm{Re}\,X_\textrm{eff} =-|X_\textrm{eff}|\cos \beta _X, \qquad \textrm{Im}\,X_\textrm{eff} =|X_\textrm{eff}|\sin \beta _X, \end{aligned}$$

Equations (8) and (9) can also be written as follows:

22 $$\begin{aligned}  &   \mathcal {B}(K^+\rightarrow \pi ^+\nu \bar{\nu })=\kappa _+ (1+\Delta _\text {EM})\cdot \nonumber \\  &   \left[ \frac{|X_\textrm{eff}|^2}{\lambda ^{10}}+2\bar{P}_c(X)|X_\textrm{eff}| \frac{\cos \beta _X}{\lambda ^5} +\bar{P}_c(X)^2\right] , \end{aligned}$$

where we introduced

23 $$\begin{aligned} \bar{P}_c(X)= - \frac{{Re}\lambda _c}{\lambda } P_c(X) = \left( 1-\frac{\lambda ^2}{2}\right) P_c(X)\,, \end{aligned}$$

and

24 $$\begin{aligned} \mathcal {B}({K_L \rightarrow \pi ^0\nu \bar{\nu }})=\kappa _L\cdot \frac{|X_\textrm{eff}|^2}{\lambda ^{10}}\sin ^2\beta _X \,. \end{aligned}$$

Alternatively, we can solve (8) and (9) for $\textrm{Re}\,X_\textrm{eff}$ and $\textrm{Im}\,X_\textrm{eff}$ [27]:

25 $$\begin{aligned} \textrm{Re}\,X_\textrm{eff}&{=} \mp \lambda ^5\left[ \frac{\mathcal{B}({K^+ \rightarrow \pi ^+\nu \bar{\nu }})}{\kappa _+(1 + \Delta _\textrm{EM})} {-} \frac{\mathcal{B}({K_L \rightarrow \pi ^0\nu \bar{\nu }})}{\kappa _L}\right] ^{1/2} \nonumber \\&\quad - \lambda ^4\textrm{Re}\,\lambda _c P_c(X)\,,\end{aligned}$$

26 $$\begin{aligned} \textrm{Im}\,X_\textrm{eff}&= \pm \lambda ^5\left[ \frac{\mathcal{B}({K_L \rightarrow \pi ^0\nu \bar{\nu }})}{\kappa _L}\right] ^{1/2}\,. \end{aligned}$$

These expressions can be used to directly determine $X_\text {eff}$ once the branching ratios of the decays ${K^+ \rightarrow \pi ^+\nu \bar{\nu }}$ and ${K_L \rightarrow \pi ^0\nu \bar{\nu }}$ have been measured. As evident from the signs ∓ and ± in front of the square-root terms, a four-fold ambiguity remains in this determination. The upper sign in both formulae corresponds to the case in which NP contributions do not reverse the sign of the SM functions. As pointed out in [50], within specific NP scenarios these ambiguities can be resolved by considering other processes, such as the non-leptonic *B* decays and the rare decays discussed in [50].^4^ Indeed any process sensitive to electroweak penguins that contribute to $X_\textrm{eff}$ can be useful in this respect. This also applies to the ratio $\varepsilon '/\varepsilon$, as seen in the explicit expressions in [51].

As pointed out in [50] it is possible to find a NP scenario in which, despite a sizeable NP contribution to $X_\text {eff}$, $\mathcal {B}({K^+ \rightarrow \pi ^+\nu \bar{\nu }})$ does not significantly differ from the SM estimate because the enhancement of the first term in (22) can be compensated by the suppression of the second term. In this way, a spectacular enhancement of $\mathcal {B}({K_L \rightarrow \pi ^0\nu \bar{\nu }})$ by one order of magnitude can still be obtained. Consequently, while $\mathcal {B}({K_L \rightarrow \pi ^0\nu \bar{\nu }})\approx (1/3)\mathcal {B}({K^+ \rightarrow \pi ^+\nu \bar{\nu }})$ in the SM, it can be substantially larger than $\mathcal {B}({K^+ \rightarrow \pi ^+\nu \bar{\nu }})$ in such a NP scenario. As illustrated already in [50] and updated below, the huge enhancement of $\mathcal {B}({K_L \rightarrow \pi ^0\nu \bar{\nu }})$ necessarily requires a large weak phase $\beta _X\approx 110^\circ$, as

27 $$\begin{aligned} \frac{\mathcal {B}({K_L \rightarrow \pi ^0\nu \bar{\nu }})}{\mathcal {B}({K_L \rightarrow \pi ^0\nu \bar{\nu }})_\textrm{SM}}= \left| \frac{X_\textrm{tot}}{X_\textrm{SM}}\right| ^2 \left[ \frac{\sin \beta _X}{\sin (\beta -\beta _s)}\right] ^2\,, \end{aligned}$$

where values of β and $\beta _{s}$ are given in Appendix A. $\mathcal {B}({K_L \rightarrow \pi ^0\nu \bar{\nu }})$ can then be rather close to the Grossman-Nir bound [28]

28 $$\begin{aligned} \mathcal{B}({K_L \rightarrow \pi ^0\nu \bar{\nu }})\le 4.3\, \mathcal {B}({K^+ \rightarrow \pi ^+\nu \bar{\nu }}). \end{aligned}$$

This model-independent upper bound on $\mathcal{B}({K_L \rightarrow \pi ^0\nu \bar{\nu }})$ holds in any NP scenario with SM neutrinos in the final state. It could, however, be violated in models in which other massive dark particles appear in the final state, provided that their effect remains hidden from the NA62 analysis due to the experimental setup [52]. Note that at the Grossman-Nir bound [28] the square root in (25) vanishes.

**Fig. 2:**
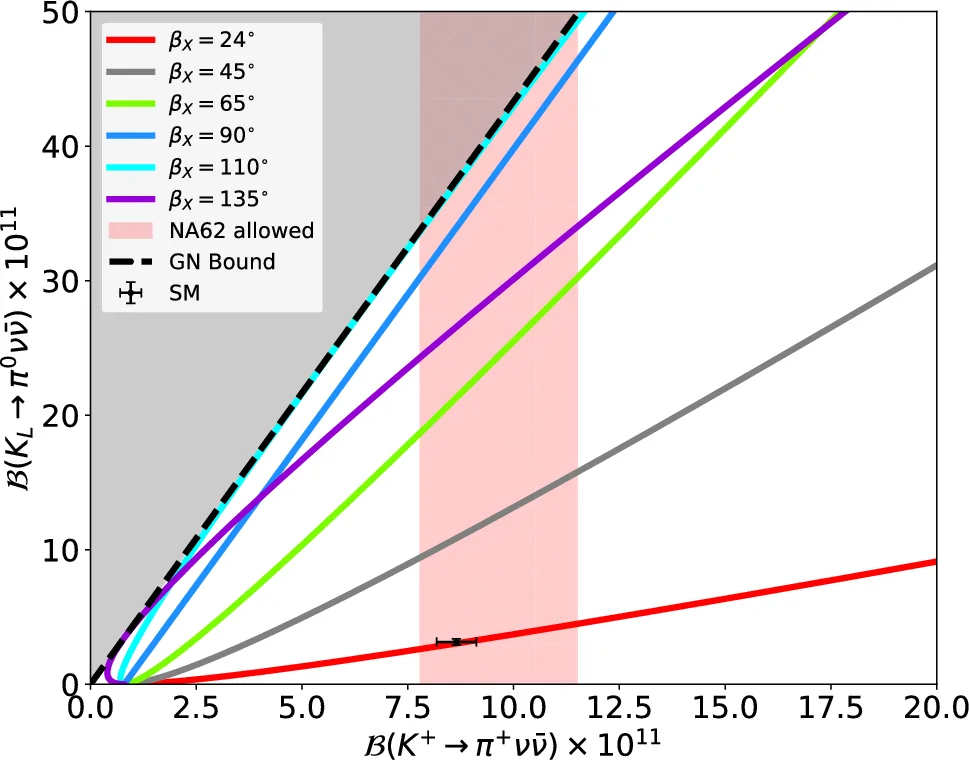
Correlation between $\mathcal {B}({K^+ \rightarrow \pi ^+\nu \bar{\nu }})$ and $\mathcal {B}({K_L \rightarrow \pi ^0\nu \bar{\nu }})$ for different values of the phase $\beta _X$

**Fig. 3:**
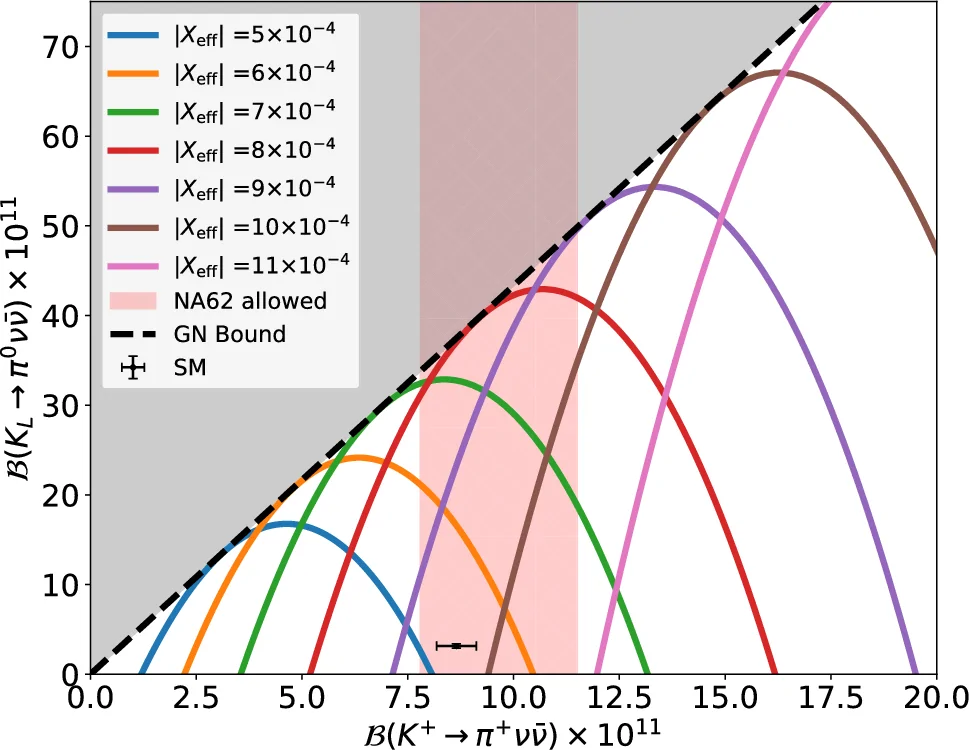
Correlation between $\mathcal {B}({K^+ \rightarrow \pi ^+\nu \bar{\nu }})$ and $\mathcal {B}({K_L \rightarrow \pi ^0\nu \bar{\nu }})$ for different values of $|X_\text {eff}|$

**Fig. 4:**
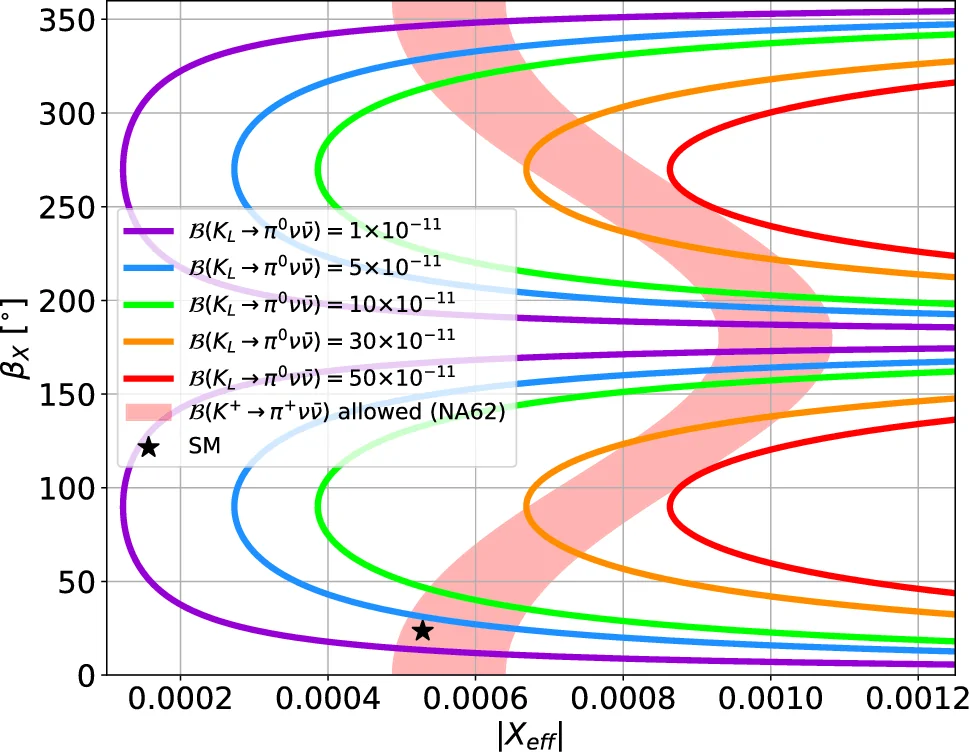
Determination of $|X_\text {eff}|$ and $\beta _X$ from the $K\rightarrow \pi \nu \bar{\nu }$ decays. The red shaded region shows the 1σ allowed range for $\mathcal {B}({K^+ \rightarrow \pi ^+\nu \bar{\nu }})$ by NA62, while the coloured lines display different hypothetical measurements of $\mathcal {B}({K_L \rightarrow \pi ^0\nu \bar{\nu }})$

Figures 2, 3 and 4 illustrate the impact of possible NP effects parametrised by the function $X_\text {eff}$ on the branching ratios of ${K^+ \rightarrow \pi ^+\nu \bar{\nu }}$ and ${K_L \rightarrow \pi ^0\nu \bar{\nu }}$. In Fig. 2, we show $\mathcal {B}({K_L \rightarrow \pi ^0\nu \bar{\nu }})$ as a function of $\mathcal {B}({K^+ \rightarrow \pi ^+\nu \bar{\nu }})$ for fixed values of $\beta _X$. The magnitude of $|X_\text {eff}|$ is varied along the lines. It is evident that the NA62 constraint, shown by the red band, implies that NP models with SM-like phases, $\beta _X \sim 24^\circ$, predict $\mathcal {B}({K_L \rightarrow \pi ^0\nu \bar{\nu }})$ to lie in the vicinity of its SM value, below $5\times 10^{-11}$.

However, the prospects for $\mathcal {B}({K_L \rightarrow \pi ^0\nu \bar{\nu }})$ change drastically when allowing for a large complex phase $\beta _X$. In particular, for values in the ballpark of $\beta _X \sim 110^\circ$ the branching ratio of ${K_L \rightarrow \pi ^0\nu \bar{\nu }}$ is predicted close to the model-independent Grossman–Nir bound [28] given in (28), shown by the black dashed line. It is worth noting that for $\beta _X = 90^\circ$, shown in blue, the two branching ratios lie on a line parallel to the Grossman-Nir bound, with the offset determined by the charm contribution to ${K^+ \rightarrow \pi ^+\nu \bar{\nu }}$.

Analogously in Fig. 3, we show $\mathcal {B}({K_L \rightarrow \pi ^0\nu \bar{\nu }})$ as a function of $\mathcal {B}({K^+ \rightarrow \pi ^+\nu \bar{\nu }})$ for fixed values of $|X_\text {eff}|$, varying the phase $\beta _X$ along the different lines. We find that the NA62 measurement implies $|X_\text {eff}|$ to lie in the range

29 $$\begin{aligned} 4.8\times 10^{-4} {\mathop {_\sim }\limits ^{<}}|X_\text {eff}| {\mathop {_\sim }\limits ^{<}}10.9 \times 10^{-4}\,. \end{aligned}$$

For larger (smaller) values of $|X_\text {eff}|$, too large (small) values for $\mathcal {B}({K^+ \rightarrow \pi ^+\nu \bar{\nu }})$ are predicted irrespective of the value of $\beta _X$. Thus the current $\mathcal {O}(20\%)$ experimental uncertainty by NA62 leads us to constrain $|X_\text {eff}|$ up to about a factor of two. Note that within the SM $|X_\text {eff}|$ reduces to $|\lambda _t X(x_t)| = 5.3 \times 10^{-4}$.

Figure 4 shows how NA62 and KOTO II can determine $|X_\textrm{eff}|$ and $\beta _X$: the red band indicates the 1σ range from NA62, while the coloured lines give the predicted ${K_L \rightarrow \pi ^0\nu \bar{\nu }}$ branching ratio as indicated by the legend. Again one clearly sees that a large CP-violating phase $\beta _X$ is required for a large ${K_L \rightarrow \pi ^0\nu \bar{\nu }}$ branching ratio, while keeping the ${K^+ \rightarrow \pi ^+\nu \bar{\nu }}$ branching ratio consistent with the recent NA62 result. It is worth noting that currently, without a measurement of $\mathcal{B}({K_L \rightarrow \pi ^0\nu \bar{\nu }})$, the value of $\beta _X$ is unconstrained in the model-independent consideration and it could take any value between 0 and $360^\circ$.

The four-fold ambiguity in the determination of $|X_\textrm{eff}|$ and $\beta _X$ from the $K\rightarrow \pi \nu \bar{\nu }$ decays, already discussed below eqs. (25) and (26), becomes evident from this figure. It is impossible to resolve it from these two decays alone. However, as we mentioned already, in specific NP scenarios other decays can help in resolving this ambiguity.

### $K_{L}\rightarrow \mu ^+\mu ^-$

The decay $K_{L}\rightarrow \mu ^+\mu ^-$ is subject to significant long-distance (LD) contributions, preventing a precise prediction of its branching ratio. Only the SD part of the dispersive contribution to $K_L\rightarrow \mu ^+\mu ^-$ can be reliably calculated. Despite this limitation, the constraint on the SD contribution places important bounds on certain NP scenarios. In contrast to ${K^+ \rightarrow \pi ^+\nu \bar{\nu }}$ and ${K_L \rightarrow \pi ^0\nu \bar{\nu }}$ (where only the vector current contributes) here the axial-vector current contributes. Relating the relevant matrix element $\langle 0|\bar{s}\gamma _\mu P_L d |K_L\rangle$ to the branching ratio $\mathcal {B}(K^+\rightarrow \mu ^+\nu _\mu )$ one finds [40]

30 $$\begin{aligned} {\mathcal {B}(K_L\rightarrow \mu ^+\mu ^-)_\textrm{SD} = \kappa _\mu \left( \frac{\textrm{Re}\,Y^A_\textrm{eff}}{\lambda ^5} + \frac{\textrm{Re}\,\lambda _c}{\lambda } P_c(Y) \right) ^2\,,} \end{aligned}$$

where

31 $$\begin{aligned}  &   \kappa _\mu = 2.01\cdot 10^{-9},\qquad \nonumber \\  &   Y(x_t)=(0.9882\pm 0.00024) Y_0(x_t), \qquad \nonumber \\  &   P_c(Y) = 0.115\pm 0.017.\nonumber \\ \end{aligned}$$

$Y(x_t)$ is the SM contribution at the NNLO level [53], where the LO Inami-Lim function is, to an excellent approximation, given by

32 $$\begin{aligned} Y_0(x_t) = 0.315 x_t^{0.78}\,, \end{aligned}$$

and $P_c(Y)$ represents the charm contribution at NNLO [54].

Similarly to $X_\textrm{eff}$, we have introduced

33 $$\begin{aligned} Y^A_\textrm{eff} = V_{ts}^* V_{td} \,(Y(x_t)+\Delta Y_A(K))=-|Y^A_\textrm{eff}|e^{-i\beta _Y} \end{aligned}$$

and

34 $$\begin{aligned} Y^A_\textrm{tot}=Y(x_t)+\Delta Y_A(K)=|Y^A_\textrm{tot}|e^{i\theta _Y} \end{aligned}$$

with $\beta _Y=\beta -\beta _s-\theta _Y$. Here the NP contribution is represented by $\Delta Y_A(K)$. For the $Z^\prime$ scenario it is given in (70).

We find then

35 $$\begin{aligned} \mathcal {B}(K_L\rightarrow \mu ^+\mu ^-)^\text {SM}_\textrm{SD} = (8.48\pm 0.73)\times 10^{-10}. \end{aligned}$$

The extraction of the SD part from data is subject to considerable uncertainties. Here the important issue is the sign of the interference of the SD dispersive part $\chi _\textrm{SD}$ of the decay amplitude of $K_L \rightarrow \mu ^+\mu ^-$ with the corresponding LD parts. Allowing for both possible signs implies a conservative bound $|\chi _\textrm{SD}| \le 3.1$ [9]. This gives then the known upper bound [9]

36 $$\begin{aligned} \mathcal {B}(K_L\rightarrow \mu ^+\mu ^-)_\textrm{SD} \le 2.5 \times 10^{-9}\,, \end{aligned}$$

roughly three times as large as the SM value. This bound is also obtained for the sign favoured in [55, 56] that implies $-1.7 \le \chi _\textrm{SD}\le 3.1$.

On the other hand the opposite sign is favoured in [57], giving $-3.1 \le \chi _\textrm{SD}\le 1.7$ and therefore approximately

37 $$\begin{aligned} \mathcal {B}(K_L\rightarrow \mu ^+\mu ^-)_\textrm{SD} \le \mathcal {B}(K_L\rightarrow \mu ^+\mu ^-)^\textrm{SM}_\textrm{SD}\,. \end{aligned}$$

It is well known that these bounds preclude large enhancements of $\mathcal {B}({K^+ \rightarrow \pi ^+\nu \bar{\nu }})$ for models with NP governed by LH currents but are much less important if RH currents dominate the NP contributions. Yet, in view of the recent NA62 result such an enhancement is no longer allowed anyway.

As already mentioned above it has been pointed out in [11] and further analysed in [58–62] that a clean extraction of the SD parameters of the decay $K\rightarrow \mu ^+\mu ^-$ is possible from a measurement of the $K_L-K_S$ interference term in the time-dependent rate. Consequently direct CP violation can be measured in this decay. This brings us to the next even more interesting decay.

### $K_{S}\rightarrow \mu ^+\mu ^-$

The decay $K_S\rightarrow \mu ^+\mu ^-$ provides a sensitive probe of imaginary parts of new $\bar{s}d$ couplings. Its branching fraction receives LD and SD contributions, which are added incoherently in the total rate [9, 63]. This is in contrast to the decay $K_L\rightarrow \mu ^+\mu ^-$, where LD and SD amplitudes interfere, and moreover $\mathcal{B}(K_L\rightarrow \mu ^+\mu ^-)$ is sensitive to the real parts of $\bar{s}d$ couplings. The SD part of $\mathcal{B}(K_S\rightarrow \mu ^+\mu ^-)$ is given as

38 $$\begin{aligned}  &   \mathcal{B}(K_S\rightarrow \mu ^+\mu ^-)_\textrm{SD}\nonumber \\  &   {=} \tau _{K_S} \frac{G_F^2 \alpha ^2}{8 \pi ^3\sin ^4\theta _W} m_K F_K^2 \sqrt{1{-}\frac{m^2_\mu }{m_K^2}} m_\mu ^2\, \left[ \textrm{Im} Y^A_\textrm{eff} \right] ^2\nonumber \\  &   =1.04\times 10^{-5}\left[ \textrm{Im} Y^A_\textrm{eff} \right] ^2, \end{aligned}$$

with $Y^A_\textrm{eff}$ given in (33).

The SM prediction [9, 11] reads

39 $$\begin{aligned}&\mathcal{B}(K_S\rightarrow \mu ^+\mu ^-)_\textrm{SM} \nonumber \\&= (4.99_\textrm{LD} + 0.19_\textrm{SD}) \times 10^{-12} = (5.2 \pm 1.5) \times 10^{-12}, \end{aligned}$$

about a factor of 50 below the present upper bound in (6). The predicted SD contribution therefore lies three orders of magnitude below the experimental bound. As already mentioned previously it has been demonstrated in [60] that the short distance contribution to $K_{S}\rightarrow \mu ^+\mu ^-$ can be extracted from data, making it another precision observable.

This is important because this decay—being dominated by direct CP violation in models with axial-vector currents—is very sensitive to NP contributions, as recently analysed in [64]. For earlier studies see in particular the analyses of $Z^\prime$ models [65], leptoquark models [66], and several models reviewed in [35].

### $K_L\rightarrow \pi ^0 \ell ^+\ell ^-$

The rare decays $K_L\rightarrow \pi ^0e^+e^-$ and $K_L\rightarrow \pi ^0\mu ^+\mu ^-$ are dominated by CP-violating contributions. KOTO II plans to have a second phase (after $K_L\rightarrow \pi ^0\nu \bar{\nu }$) to measure $K_L\rightarrow \pi ^0e^+e^-$ (the di-muon channel is also under investigation but is more challenging experimentally) [8].

In the SM the main contribution comes from the indirect (mixing-induced) CP violation and its interference with the direct CP-violating contribution [67–70]. The direct CP-violating contribution to the branching ratio is within the SM in the ballpark of $4\times 10^{-12}$, while the CP conserving contribution is at most $3\times 10^{-12}$. Among the rare *K* meson decays, the decays in question are among the theoretically cleanest, but certainly cannot compete with the $K\rightarrow \pi \nu \bar{\nu }$ decays. Moreover, the dominant indirect CP-violating contributions are practically determined by the measured decays $K_S\rightarrow \pi ^0\ell ^+\ell ^-$ and the parameter $\varepsilon _K$. Consequently they are not as sensitive as the $K_L\rightarrow \pi ^0\nu \bar{\nu }$ decay to NP contributions, present only in the subleading direct CP violation. However, in the presence of large new CP-violating phases, the direct CP-violating contribution can become the dominant contribution and the branching ratios for $K_L\rightarrow \pi ^0\ell ^+\ell ^-$ can be enhanced significantly, with a stronger effect in the case of $K_L\rightarrow \pi ^0\mu ^+\mu ^-$ as already analysed in [69–71]. Yet, even more important are the correlations of these decays with ${K_L \rightarrow \pi ^0\nu \bar{\nu }}$ and the ratio $\varepsilon '/\varepsilon$ which we will encounter in Sect. 4.

In analogy to $X_\textrm{eff}$ and $Y^A_\textrm{eff}$, we have in these decays^5^

40 $$\begin{aligned} Y^V_\textrm{eff}= &   V_{ts}^* V_{td} \,(Y_0(x_t)+\Delta Y_V(K))=-|Y^V_\textrm{eff}|e^{-i\bar{\beta }_Y},\end{aligned}$$

41 $$\begin{aligned} Y^V_\textrm{tot}= &   Y_0(x_t)+\Delta Y_{V}(K)=|Y^V_\textrm{tot}|e^{i\bar{\theta }_Y}, \end{aligned}$$

and

42 $$\begin{aligned} Z^V_\textrm{eff}= &   V_{ts}^* V_{td} \,(Z_0(x_t)+\Delta Z_V(K))=- |Z^V_\textrm{eff}|e^{-i\beta _Z} ,\end{aligned}$$

43 $$\begin{aligned} Z^V_\textrm{tot}= &   Z_0(x_t)+\Delta Z_{V}(K)=|Z^V_\textrm{tot}|e^{i\theta _Z}. \end{aligned}$$

For the $Z^\prime$ scenario $\Delta Y_{V}(K)$ and $\Delta Z_{V}(K)$ are given in (72) and (73), respectively. It should be noticed that in these decays even at the NLO level QCD corrections do not affect the SM one-loop functions $Y_0(x_t)$ and $Z_0(x_t)$ which for $m_t = 162.69\,\text {GeV}$ are given by

44 $$\begin{aligned} Y_0(x_t)=0.315 x_t^{0.78},\qquad Z_0(x_t)= 0.175 x_t^{0.93}. \end{aligned}$$

Only the $m_t$ independent contribution $P_0$ is affected by QCD and given at the NLO level by $P_0=2.88\pm 0.06$ [72].

We have then [69–71]

45 $$\begin{aligned} \mathcal {B}(K_L\rightarrow \pi ^0\ell ^+\ell ^-)&{=}\left( C_\text {dir}^\ell \pm C_\text {int}^\ell \left| a_s\right| {+} C_\text {mix}^\ell \left| a_s\right| ^2{+}C_\text {CPC}^\ell \right) \nonumber \\&\quad \times 10^{-12}\,, \end{aligned}$$

where

46 $$\begin{aligned}&C_\text {dir}^e = (4.62\pm 0.24)(\omega _{7V}^2+\omega _{7A}^2)\,, \nonumber \\&C_\text {dir}^\mu =(1.09\pm 0.05)(\omega _{7V}^2+2.32\omega _{7A}^2)\,,\end{aligned}$$

47 $$\begin{aligned}&C_\text {int}^e = (11.3\pm 0.3)\omega _{7V}\,, \nonumber \\&C_\text {int}^\mu = (2.63\pm 0.06)\omega _{7V}\,,\end{aligned}$$

48 $$\begin{aligned}&C_\text {mix}^e = 14.5\pm 0.05\, \nonumber \\&C_\text {mix}^\mu = 3.36\pm 0.20\,,\end{aligned}$$

49 $$\begin{aligned}&C_\text {CPC}^e \simeq 0\, \nonumber \\&C_\text {CPC}^\mu = 5.2\pm 1.6\,,\end{aligned}$$

50 $$\begin{aligned}&\left| a_s\right| =1.2\pm 0.2 \end{aligned}$$

with

51 $$\begin{aligned} \omega _{7V}= &   \frac{1}{2\pi }\Bigg [P_0+\frac{|Y^V_\textrm{tot}|}{\sin ^2\theta _W} \frac{\sin \bar{\beta }_Y}{\sin (\beta -\beta _s)}\nonumber \\  &   -4|Z^V_\textrm{tot}| \frac{\sin \beta _Z}{\sin (\beta -\beta _s)}\Bigg ]\left[ \frac{\text {Im}\,\lambda _t}{1.4\cdot 10^{-4}}\right] ,\end{aligned}$$

52 $$\begin{aligned} \omega _{7A}= &   -\frac{1}{2\pi }\frac{|Y^V_\textrm{tot}|}{\sin ^2\theta _W} \frac{\sin \bar{\beta }_Y}{\sin (\beta -\beta _s)}\left[ \frac{\text {Im}\,\lambda _t}{1.4\cdot 10^{-4}}\right] , \end{aligned}$$

where

53 $$\begin{aligned} \bar{\beta }_Y=\beta -\beta _s-\bar{\theta }_Y\,,\qquad \beta _Z=\beta -\beta _s-\theta _Z \end{aligned}$$

with $Z^V_\textrm{tot}$ defined in (43).

The present experimental bounds

54 $$\begin{aligned} & {\mathcal {B}}(K_L\rightarrow \pi ^0e^+e^-)<28\times 10^{-11}\quad \text {[13]}\,,\nonumber \\ &  {\mathcal {B}}(K_L\rightarrow \pi ^0\mu ^+\mu ^-)<38\times 10^{-11}\quad \text {[14]} \end{aligned}$$

are still roughly one order of magnitude larger than the SM predictions, which, using the CKM parameters of Appendix A, we find to be

55 $$\begin{aligned} \mathcal {B}(K_L\rightarrow \pi ^0e^+e^-)_\text {SM}&{=} 3.51\pm 0.86 \left( 1.56\pm 0.54\right) \times 10^{-11}\,, \end{aligned}$$

56 $$\begin{aligned} {\mathcal {B}(K_L\rightarrow \pi ^0\mu ^+\mu ^-)_\text {SM}}&{=} {1.40\pm 0.26 \left( 0.95\pm 0.20\right) \times 10^{-11}}\,, \end{aligned}$$

with the values in parentheses corresponding to the “−” sign in (45). The central values of these predictions are very close to the original ones in [71] but the uncertainties decreased.

### $\varepsilon '/\varepsilon$

The parameter $\varepsilon '/\varepsilon$ measures the amount of direct CP violation in $K\rightarrow \pi \pi$ decays. Its experimental world average, obtained by combining the results from the NA48 [17] and KTeV [18, 73] collaborations, reads

57 $$\begin{aligned} {(\varepsilon '/\varepsilon )_\text {exp} = (16.6 \pm 2.3) \times 10^{-4}\,.} \end{aligned}$$

A short description of the present status of $\varepsilon '/\varepsilon$ within the SM is given in [25]. For our purposes it is sufficient to state that it is unclear at present whether the estimate of $\varepsilon '/\varepsilon$ in the SM agrees with the result in (57).

Indeed, the most recent result from lattice QCD, by the RBC-UKQCD collaboration [21], reads

58 $$\begin{aligned} (\varepsilon '/\varepsilon )_\textrm{SM}^\text {LQCD} = (21.7 \pm 8.4) \times 10^{-4} \,, \end{aligned}$$

where statistical, parametric, and systematic uncertainties have been added in quadrature. On the other hand the most recent estimate of $\varepsilon '/\varepsilon$ in the SM from ChPT [22, 23] reads

59 $$\begin{aligned} (\varepsilon '/\varepsilon )_\text {SM}^\text {ChPT} = (14 \pm 5) \times 10^{-4} \,. \end{aligned}$$

Both results are consistent with the experimental result in (57), but the large uncertainties still allow significant NP contributions.

Finally, based on the insight from Dual QCD [20, 74] one finds [24]

60 $$\begin{aligned} (\varepsilon '/\varepsilon )_\textrm{SM}^\text {DQCD}= (5\pm 2)\times 10^{-4}\,, \end{aligned}$$

which, if true, would be one of the largest anomalies in flavour physics.

We will not repeat the arguments of the second author [24] why he thinks that the last result, even if rather uncertain, is closest to the true SM result among the three results quoted here. Instead we simply write

61 $$\begin{aligned} \frac{\varepsilon '}{\varepsilon }=\left( \frac{\varepsilon '}{\varepsilon }\right) ^\textrm{SM}+\left( \frac{\varepsilon '}{\varepsilon }\right) ^\textrm{NP}\,, \qquad \left( \frac{\varepsilon '}{\varepsilon }\right) ^\textrm{NP}= \kappa _{\varepsilon ^\prime }\cdot 10^{-3}, \end{aligned}$$

and, in view of the large uncertainties present in the SM estimates of $\varepsilon '/\varepsilon$, we concentrate fully on the contribution of NP. In particular, we will investigate whether a positive shift in $\varepsilon '/\varepsilon$ with $\kappa _{\varepsilon ^\prime }=\mathcal {O}(1)$ can be generated by a $Z^\prime$ contribution.

## Specific $\boldsymbol{Z^\prime }$ scenario

### Preface

An important class of models that can generate large NP contributions to rare meson decays are $Z^\prime$ models, in which the SM is accompanied by a new neutral vector boson related to a new $U(1)^\prime$ gauge symmetry. Within $Z^\prime$ models, the same flavour-changing quark couplings that enter ΔF=1 flavour-changing neutral currents (FCNCs) also give rise to tree-level contributions to ΔF=2 meson mixing observables. As these can be measured with high precision, they impose stringent constraints on the parameter space of $Z^\prime$ models and limit their potential effects in FCNCs.

However, as emphasised in [75–77], the NP contributions to ΔF=2 processes are suppressed for a particular pattern of left-handed $\Delta _L^{q_1 q_2}(Z^\prime )$ and right-handed $\Delta _R^{q_1 q_2}(Z^\prime )$ flavour-violating $Z^\prime$ couplings to quarks $q_1$ and $q_2$ defined through62As stressed in particular in [75], in the case of the decays considered by us this implies that the $\Delta _R^{sd}(Z^\prime )$ coupling must be roughly two orders of magnitude smaller than $\Delta _L^{sd}(Z^\prime )$ in order to keep $\varepsilon _K$ consistent with experiment. We will be more explicit about this in the next section. As we shall see, this simplifies our analysis significantly while allowing $\Delta _L^{sd}(Z^\prime )$ to be sufficiently large to provide significant NP contributions to the rare kaon decays and the ratio $\varepsilon '/\varepsilon$ considered by us.

A compendium of the $Z^\prime$ contributions to the observables considered by us has been presented in [65] and can also be found in Section 15.4 of [35]. From these references, we extract the expressions used in the present paper. Note that we still keep $\Delta _R^{sd}(Z^\prime )$ in these expressions in order to make explicit the simplifications resulting from its smallness.

### $\varepsilon _K$ and $\Delta M_K$

**Fig. 5:**
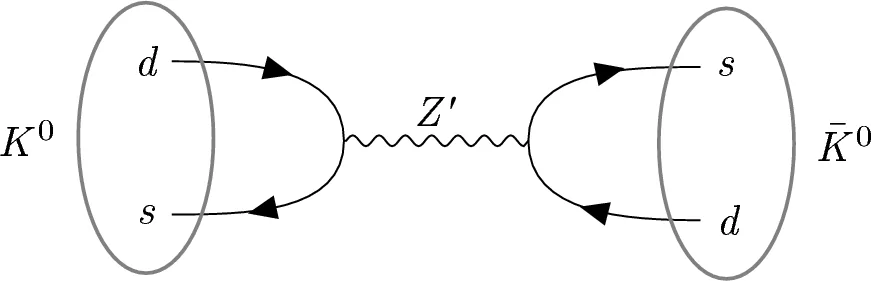
Feynman diagram for $$K^0-\bar{K}^0$$ mixing in $Z^{\prime }$ models

The contribution of a $Z^\prime$ boson to the parameter $\varepsilon _K$, measuring CP violation in $K^0-\bar{K}^0$ mixing, and the mass difference $\Delta M_K$ are depicted in Fig. 5 and given as follows

63 $$\begin{aligned}  &   \varepsilon _K(Z^\prime )=\frac{\kappa _\varepsilon e^{i\phi _\varepsilon }}{\sqrt{2}(\Delta M_K)_\textrm{exp}} \text {Im}\left( (M_{12})_{Z^\prime }^{sd}\right) , \qquad \nonumber \\  &   \Delta M_K(Z^\prime )= 2\text {Re}\left( (M_{12})_{Z^\prime }^{sd}\right) , \end{aligned}$$

where $\kappa _\epsilon = 0.94(2)$ [78, 79]. They should simply be added to the SM contributions.

In the presence of both LH and RH couplings of a $Z^\prime$ gauge boson to SM quarks, the left-right (LR) ΔF=2 operators are generated, whose contributions to the mixing amplitude $M_{12}^{sd}$ are enhanced through renormalisation group effects relative to left-left (VLL) and right-right (VRR) operators. Moreover, in the case of $M_{12}^{sd}$ additional chiral enhancements of the hadronic matrix elements of LR operators are present. As pointed out in [75] this fact can be used to suppress NP contributions to $\varepsilon _K$ and $\Delta M_K$ through some fine-tuning between VLL, VRR and LR contributions, thereby allowing for larger contributions to other observables.

This idea, used in [76], is also very useful for our present analysis. To this end, one writes the $Z^\prime$ contributions to the mixing amplitude as follows [65]:

64 $$\begin{aligned} (M_{12}^*)_{Z^\prime }^{sd} = \frac{\left( \Delta _L^{sd}(Z')\right) ^2}{2M_{Z^\prime }^2} \langle \hat{Q}_1^\text {VLL}(M_{Z^\prime })\rangle ^{sd} z_{sd}, \end{aligned}$$

where $z_{sd}$ is complex and given by

65 $$\begin{aligned}  &   z_{sd}=\left[ 1+\left( \frac{\Delta _R^{sd}(Z')}{\Delta _L^{sd}(Z')}\right) ^2+2\kappa _{sd}\frac{\Delta _R^{sd}(Z')}{\Delta _L^{sd}{Z')}}\right] , \nonumber \\  &   \qquad \kappa _{sd}=\frac{\langle \hat{Q}_1^\text {LR}(M_{Z^\prime })\rangle ^{sd}}{\langle \hat{Q}_1^\text {VLL}(M_{Z^\prime })\rangle ^{sd}}. \end{aligned}$$

Following [76], and using the technology of [80, 81], we have expressed $z_{sd}$ in terms of the renormalisation-scheme-independent matrix elements $\langle \hat{Q}_1^\text {VLL}(M_{Z^\prime })\rangle ^{sd}$ and $\langle \hat{Q}_1^\text {LR}(M_{Z^\prime })\rangle ^{sd}$, which are defined in eqs. (35) and (36) of [76]. Table 5 of [76] lists the values of these matrix elements for different $M_{Z^\prime }$. For $M_{Z^\prime }=5\, \textrm{TeV}-20\, \textrm{TeV}$ one finds $\kappa _{sd}(M_{Z^\prime })\approx -120$, so that the requirement $z_{sd}\approx 0$ implies

66 $$\begin{aligned} \frac{\Delta _R^{sd}(Z')}{\Delta _L^{sd}(Z')} \approx \frac{1}{240}. \end{aligned}$$

We thus conclude that, in order to suppress $Z^{\prime }$ contributions to $$K^0-\bar{K}^0$$ mixing by means of Eq. (66), the only role of the $$\Delta _R^{sd}(Z')$$ coupling is to keep $$\varepsilon _K$$ and $$\Delta M_{K}$$ SM-like without any impact on other observables. This in turn simplifies our numerical analysis, as will be evident in the formulae below.

### ${K^+ \rightarrow \pi ^+\nu \bar{\nu }}$ and ${K_L \rightarrow \pi ^0\nu \bar{\nu }}$

**Fig. 6:**
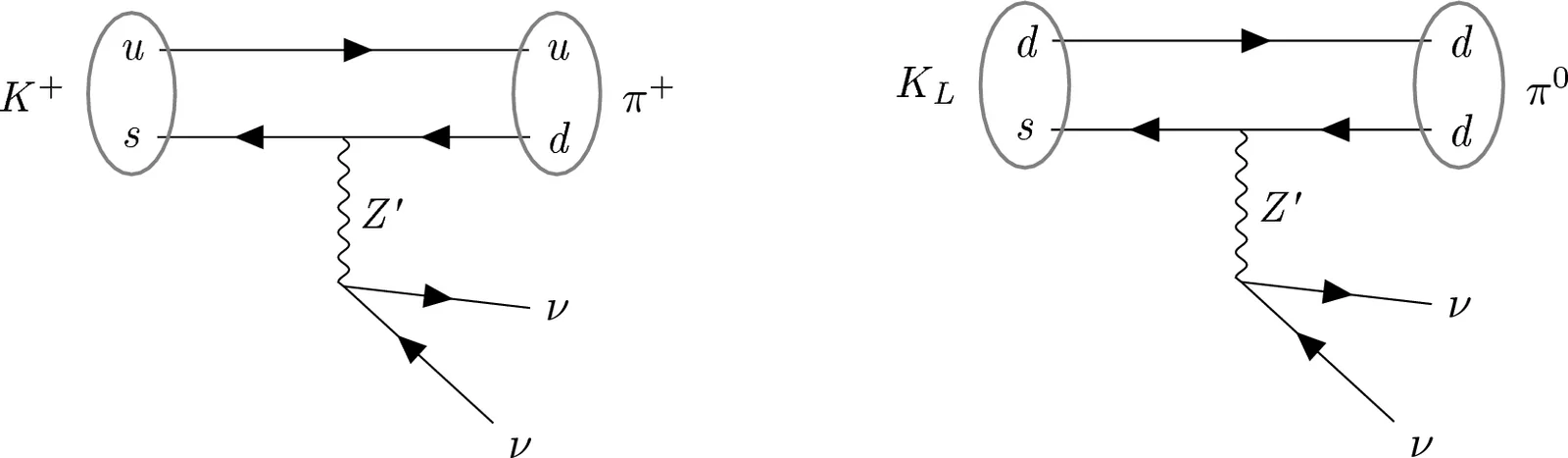
Feynman diagrams for $${K^+ \rightarrow \pi ^+\nu \bar{\nu }}$$ (left) and $${K_L \rightarrow \pi ^0\nu \bar{\nu }}$$ (right) in $Z^{\prime }$ models

The Feynman diagrams for a $Z^{\prime }$ exchange contributing to $${K^+ \rightarrow \pi ^+\nu \bar{\nu }}$$ and $${K_L \rightarrow \pi ^0\nu \bar{\nu }}$$ can be found in Fig. 6. They imply contributions to the expressions collected in Sect. 2.1 as follows:

67 $$\begin{aligned} \Delta X_{L}(K)=\frac{\Delta _L^{\nu \bar{\nu }}(Z')}{g^2_\textrm{SM}M_{Z'}^2} \frac{\Delta _L^{sd}(Z')}{V_{ts}^* V_{td}} \quad X_{R}(K)=\frac{\Delta _L^{\nu \bar{\nu }}(Z')}{g^2_\textrm{SM}M_{Z'}^2} \frac{\Delta _R^{sd}(Z')}{V_{ts}^* V_{td}} \end{aligned}$$

and therefore

68 $$\begin{aligned} X_\textrm{eff} = \lambda _{t}X(x_{t}) + \frac{1}{g_\text {SM}^{2}M_{Z^{\prime }}^{2}}\Delta _{L}^{\nu \bar{\nu }} \left( \Delta _{L}^{sd}(Z^{\prime }) + \Delta _{R}^{sd}(Z^{\prime }) \right) \,. \end{aligned}$$

We further introduced

69 $$\begin{aligned} g_{\text {SM}}^2=4\frac{G_F}{\sqrt{2}}\frac{\alpha }{2\pi \sin ^2\theta _W}=1.78137\times 10^{-7} \, \textrm{GeV}^{-2}\,. \end{aligned}$$

In what follows we neglect the small $\Delta _R^{sd}(Z^\prime )$ contribution in view of (66), so that $X_{R}(K)=0$.

### $K_{L}\rightarrow \mu ^+\mu ^-$, $K_{S}\rightarrow \mu ^+\mu ^-$ and $K_L\rightarrow \pi ^0\ell ^+\ell ^-$

**Fig. 7:**
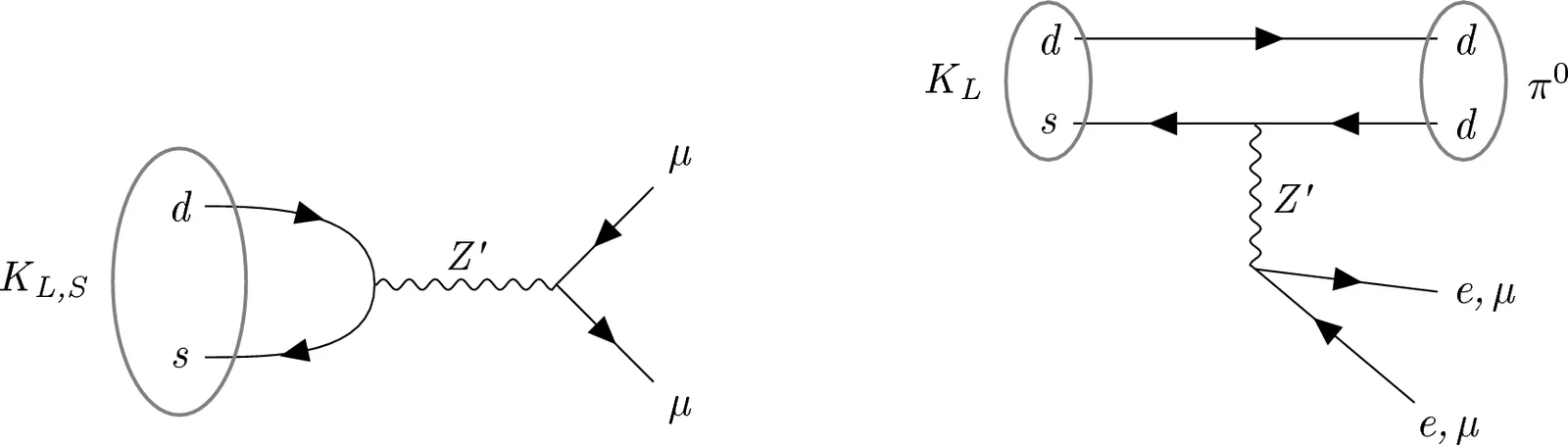
Feynman diagrams for $$K_{L,S}\rightarrow \mu ^+\mu ^-$$ (left) and $$K_L\rightarrow \pi ^0\ell ^+\ell ^-$$ (right) in $Z^{\prime }$ models

The Feynman diagrams for a $Z^{\prime }$ exchange contributing to the decays $$K_{L,S}\rightarrow \mu ^+\mu ^-$$ and $$K_L\rightarrow \pi ^0\ell ^+\ell ^-$$ can be found in Fig. 7.

For the leptonic modes $K_{L}\rightarrow \mu ^+\mu ^-$ and $K_{S}\rightarrow \mu ^+\mu ^-$ the relevant contribution to the expressions collected in Sects. 2.2 and 2.3 is given by

70 $$\begin{aligned} \Delta Y_{A}(K)=\frac{\Delta _A^{\mu \bar{\mu }}(Z')}{g^2_\textrm{SM}M_{Z'}^2} \frac{(\Delta _L^{sd}(Z')-\Delta _R^{sd}(Z'))}{V_{ts}^* V_{td}} \end{aligned}$$

and therefore

71 $$\begin{aligned} Y^{A}_\textrm{eff} = \lambda _{t}Y_{0} + \frac{1}{g_\text {SM}^{2}M_{Z^{\prime }}^{2}}\Delta _{A}^{\mu \bar{\mu }} \left( \Delta _{L}^{sd}(Z^{\prime }) - \Delta _{R}^{sd}(Z^{\prime }) \right) \,, \end{aligned}$$

where we can again set $\Delta _R^{sd}(Z^\prime )\approx 0$ in view of (66).

For the semileptonic $K_L\rightarrow \pi ^0\ell ^+\ell ^-$ modes discussed in Sect. 2.4 one finds [29, 65, 82]

72 $$\begin{aligned} \Delta Y_\textrm{V}(K)=\frac{\Delta _A^{\mu \bar{\mu }}(Z')}{g^2_\textrm{SM}M_{Z^\prime }^2} \frac{\Delta _V^{sd}(Z')}{V_{ts}^* V_{td}} \end{aligned}$$

and

73 $$\begin{aligned} \Delta Z_{V}(K)=\frac{1}{4\sin ^2\theta _W}\frac{2\Delta _R^{\mu \bar{\mu }}(Z^\prime )}{g^2_\textrm{SM}M_{Z'}^2} \frac{\Delta _V^{sd}(Z^\prime )}{V_{ts}^* V_{td}} \end{aligned}$$

with

74 $$\begin{aligned}  &   \Delta _A^{\mu \bar{\mu }}(Z')=\Delta _R^{\mu \bar{\mu }}(Z')-\Delta _L^{\mu \bar{\mu }}(Z'), \qquad \nonumber \\  &   \Delta _V^{sd}(Z')=\Delta _R^{sd}(Z')+\Delta _L^{sd}(Z')\approx \Delta _L^{sd}(Z'). \end{aligned}$$

Again in view of (66) we neglected the small contributions from $\Delta _R^{sd}(Z')$.

### $\varepsilon '/\varepsilon$

Due to the large uncertainties present in the SM estimates of $\varepsilon '/\varepsilon$, discussed in Sect. 2.5, we concentrate on the NP contribution characterised by the parameter $\kappa _{\varepsilon ^\prime }$ defined in (61). As demonstrated in [83], the NP contribution of $Z^\prime$ models is dominated by the QCD penguin operator $Q_6$ and the electroweak penguin operator $Q_8$. However, as demonstrated in [84], the $Q_6$ scenario is ruled out by significant contributions to $\varepsilon _K$ implied by renormalisation-group effects. The latter is not the case in the $Q_8$ scenario. Consequently, the NP contribution to $\varepsilon '/\varepsilon$ in our $Z^\prime$ scenario is dominated by the electroweak penguin operator

75 $$\begin{aligned} Q_8 = \frac{3}{2}\,(\bar{s}_{\alpha } d_{\beta })_{V-A}\!\!\sum _{q=u,d,s,c,b,t} e_q\,(\bar{q}_{\beta } q_{\alpha })_{V+A} \,. \end{aligned}$$

Within 10% precision it can be written as follows [83]:

76 $$\begin{aligned} \left( \frac{\varepsilon '}{\varepsilon }\right) ^\textrm{NP}=\left( \frac{\varepsilon '}{\varepsilon }\right) ^L_{Z^\prime }&= 14.5 \,B_8^{(3/2)}\,\left[ \frac{5\, \textrm{TeV}}{M_{Z^\prime }}\right] ^2 \nonumber \\&\quad {\text {Im}}\left( \Delta _L^{s d}(Z^\prime )\right) \Delta _R^{q \bar{q}}(Z^\prime )\,. \end{aligned}$$

**Fig. 8:**
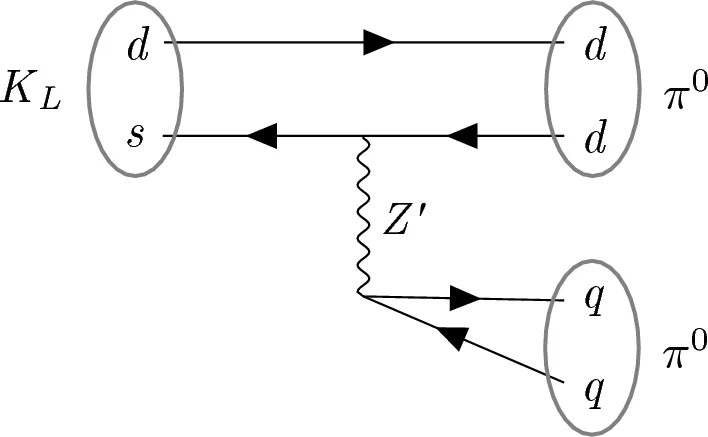
Representative Feynman diagram for $$\varepsilon '/\varepsilon $$ in the $Z^{\prime }$ scenario

This accuracy is sufficient for our analysis, not only because of the non-perturbative uncertainties in the SM contribution, but also because of the new coupling parameter $\Delta _R^{q \bar{q}}(Z^\prime )$ entering the game. In view of the smallness of the coupling $\Delta _R^{s d}(Z^\prime )$, see (66), the contribution of $Q^\prime _8$ is also negligible. A representative Feynman diagram is shown in Fig. 8.

Note that in writing (76) we have introduced the real coupling parameter $$\Delta _R^{q \bar{q}}(Z^\prime )$$ as the effective flavour-conserving $Z^{\prime }$ coupling to RH quarks entering the electroweak penguin contribution to $$\varepsilon '/\varepsilon $$, while ensuring the dangerous QCD penguin contribution to be small. See Section 4 of [84] for details.

In what follows we will set the non-perturbative factor $B_8^{(3/2)}=0.80$ as this value is consistent with both the Dual QCD approach [20] and the lattice QCD result by the RBC-UKQCD collaboration [21].

It should be remembered that, although there is considerable freedom in the values of the diagonal quark and lepton couplings of $Z^\prime$, one must ensure that they are consistent with the relevant LEP II and LHC bounds. Concerning the latter, the study in [85] implies

77 $$\begin{aligned} \left| \Delta _R^{q \bar{q}}(Z^\prime ) \right| \le 1.7 \left[ \frac{M_{Z^\prime }}{5\, \textrm{TeV}} \right] \left[ 1 + \left( \frac{1.3\, \textrm{TeV}}{M_{Z^\prime }}\right) ^2\right] \,. \end{aligned}$$

This constraint is easily satisfied by the values of $\Delta _R^{q \bar{q}}(Z^\prime )$ used in our numerical analysis. On the other hand, bounds on the leptonic $Z^\prime$ couplings can be extracted from the final analysis of the LEP-II data [86]. Yet, there is still considerable freedom, as those bounds are for products of electron and other lepton couplings. Still, to respect $\text {SU(2)}_L$ symmetry, one has to ensure that $\Delta ^{\nu \bar{\nu }}_L(Z^\prime )=\Delta ^{e\bar{e}}_L(Z^\prime )$. Furthermore, lepton universality implies $\Delta ^{e\bar{e}}_{L,R}(Z^\prime ) = \Delta ^{\mu \bar{\mu }}_{L,R}(Z^\prime )$.

## Numerical analysis for $\boldsymbol{Z^\prime }$ scenario

The simple $Z^\prime$ scenario considered in the present paper is described by the following

- four real parameters
  78 $$\begin{aligned} \Delta _L^{\nu \bar{\nu }}(Z^\prime ) = \Delta _L^{\ell \bar{\ell }}(Z^\prime ), \qquad \Delta _R^{\ell \bar{\ell }}(Z^\prime ),\qquad \Delta ^{q\bar{q}}_R(Z^\prime ),\qquad M_{Z^\prime } , \end{aligned}$$
- and two complex parameters
  79 $$\begin{aligned} \Delta _L^{sd}(Z^\prime ), \qquad \Delta _R^{sd}(Z^\prime ), \end{aligned}$$

which amounts to eight real parameters in total. Note that we have assumed $Z^{\prime }$ to be a $${U(1)^{\prime }}$$ gauge boson with lepton-flavour-universal couplings.

For our numerical analysis we choose $M_{Z^{\prime }}=5\,\text {TeV}$ and, from (66) that demonstrates $\Delta _{R}^{sd}(Z^{\prime })\ll \Delta _{L}^{sd}(Z^{\prime })$, we set $\Delta _{R}^{sd}(Z^{\prime })=0$. This reduces our parameter space to three real and one complex parameter(s),

80 $$\begin{aligned} \Delta _L^{\ell \bar{\ell }}(Z^\prime ), \qquad \Delta _R^{\ell \bar{\ell }}(Z^\prime ) , \qquad \Delta ^{\bar{q} q}_R(Z^\prime ), \qquad \Delta _L^{sd}(Z^\prime ), \end{aligned}$$

leaving us with a five-dimensional parameter space to explore.

In our numerical analysis we investigate the following quantities:

81 $$\begin{aligned} R_{\nu \bar{\nu }}^+= &   \frac{\mathcal {B}(K^+ \rightarrow \pi ^+ \nu \bar{\nu })}{\mathcal {B}(K^+ \rightarrow \pi ^+ \nu \bar{\nu })_\text {SM}} , \quad \nonumber \\ R_{\nu \bar{\nu }}^0= &   \frac{\mathcal {B}(K_L \rightarrow \pi ^0 \nu \bar{\nu })}{\mathcal {B}(K_L \rightarrow \pi ^0 \nu \bar{\nu })_\text {SM}} , \quad \nonumber \\ R_{\mu ^+\mu ^-}^L= &   \frac{\mathcal {B}(K_L \rightarrow \mu ^+\mu ^-)_\textrm{SD}}{\mathcal {B}(K_L\rightarrow \mu ^+\mu ^-)^\text {SD}_\text {SM}} , \nonumber \\ R_{\mu ^+\mu ^-}^S= &   \frac{\mathcal {B}(K_S \rightarrow \mu ^+\mu ^-)_\textrm{SD}}{\mathcal {B}(K_S \rightarrow \mu ^+\mu ^-)^\text {SD}_\text {SM}} ,\quad \nonumber \\ R^{0}_{\pi \ell ^{+}\ell ^{-}}= &   \frac{\mathcal {B}(K_L \rightarrow \pi ^0 \ell ^+ \ell ^-)}{\mathcal {B}(K_L \rightarrow \pi ^0 \ell ^+ \ell ^-)_\text {SM}} ,\quad \nonumber \\ \kappa _{\varepsilon ^{\prime }}= &   10^{3} \times \left( \frac{\varepsilon '}{\varepsilon }\right) ^\textrm{NP}. \end{aligned}$$

The dependencies of these quantities of interest, given in Eq. (81), on the parameters listed in (80) are summarised in Table 1.

**Table 1:** Functional dependencies of each variable, assuming a fixed value of $M_{Z}^{\prime }=5\,\text {TeV}$, $\Delta _{R}^{sd}(Z^{\prime })\approx 0$ and flavour-universal couplings to leptons

Variable	$\Delta _{L}^{\ell \bar{\ell }}(Z^{\prime })$	$\Delta _{R}^{\ell \bar{\ell }}(Z^{\prime })$	$\Delta _{R}^{q\bar{q}}(Z^{\prime })$	$\text {Re}\left( \Delta _{L}^{sd}(Z^{\prime })\right)$	$\text {Im}\left( \Delta _{L}^{sd}(Z^{\prime })\right)$
$R_{\nu \bar{\nu }}^{+}$	✓	–	–	✓	✓
$R_{\nu \bar{\nu }}^{0}$	✓	–	–	–	✓
$R_{\mu ^+{\mu }^-}^{L}$	✓	✓	–	✓	–
$R_{\mu ^+{\mu ^-}}^{S}$	✓	✓	–	–	✓
$R^{0}_{\pi \ell ^{+}\ell ^{-}}$	✓	✓	–	–	✓
$\kappa _{\varepsilon ^{\prime }}$	–	–	✓	–	✓

With this explicit setup, we want to demonstrate that the following set of requirements can be satisfied simultaneously:

1. The $K^0-\bar{K}^0$ mixing parameters $\varepsilon _K$ and $\Delta M_K$ are SM-like.
2. $\mathcal {B}(K^{+}\rightarrow \pi ^{+}\nu \bar{\nu })$ is consistent with the SM, and within the experimentally-allowed range from the latest NA62 measurement.
3. $\mathcal {B}(K_{L}\rightarrow \pi ^{0}\nu \bar{\nu })$, to be measured by KOTO II, can be significantly enhanced by a factor of $\mathcal {O}(10)$ with respect to its SM value.
4. The upper bound on the SD contribution to $K_L\rightarrow \mu ^+\mu ^-$ can be satisfied.
5. $\mathcal {B}(K_S\rightarrow \mu ^+\mu ^-)$, searched for by LHCb, can be significantly enhanced with respect to its SM value.
6. The branching ratios of the decays $K_L\rightarrow \pi ^0\ell ^+\ell ^-$ can be significantly enhanced, while satisfying the experimental upper limits.
7. $\varepsilon '/\varepsilon$ can be significantly enhanced with respect to its SM value, with $\kappa _{\varepsilon ^{\prime }}=\mathcal {O}(1)$.

Point 1 on this list is satisfied by our choice $\Delta _{R}^{sd}(Z^{\prime }) \approx (1/240) \Delta _{L}^{sd}(Z^{\prime })\approx 0$ which ensures $\varepsilon _K$ and $\Delta M_K$ are SM-like, regardless of the values of the other parameters.

**Fig. 9:**
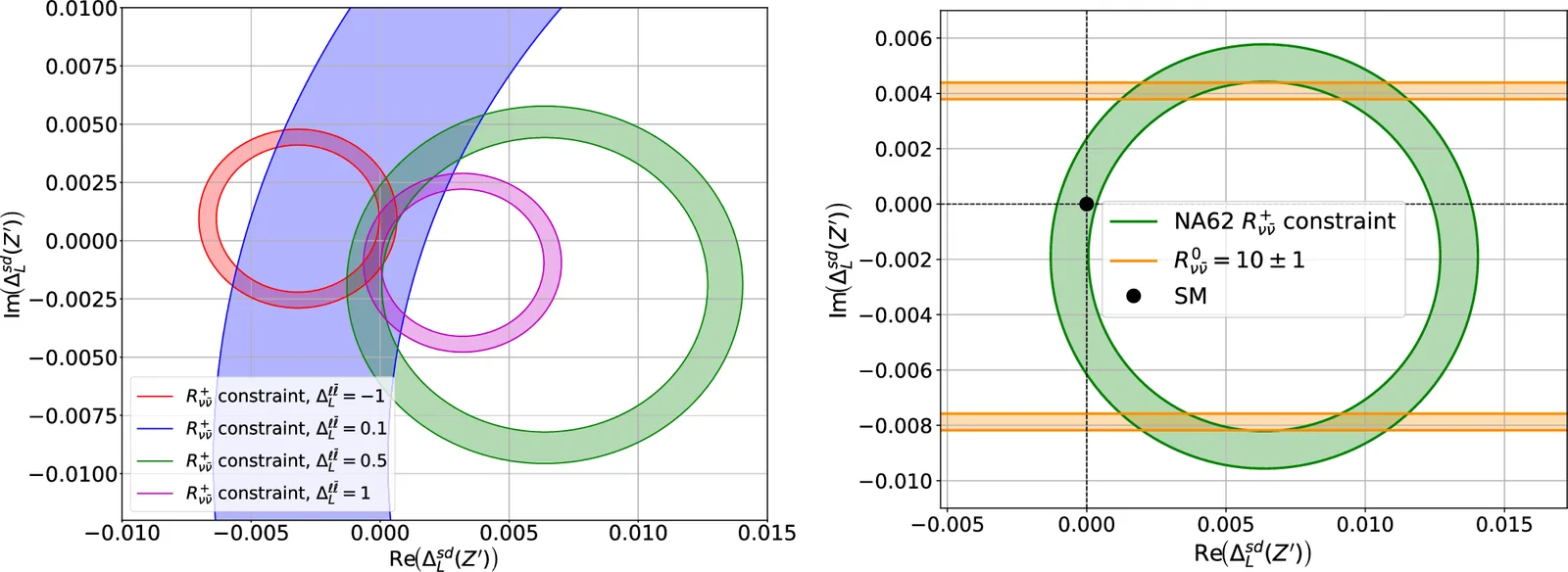
left: Constraints on $R_{\nu \bar{\nu }}^{+}$ and $R_{\nu \bar{\nu }}^{0}$ in the plane of $\left( \text {Re}\left( \Delta _{L}^{sd}(Z^{\prime })\right) , \text {Im}\left( \Delta _{L}^{sd}(Z^{\prime })\right) \right)$ for different choices of $\Delta _{L}^{\ell \bar{\ell }}(Z')$. The shaded regions correspond to the allowed 1σ interval of the NA62 measurement. Right: Assuming $\Delta _{L}^{\ell \bar{\ell }}(Z')=0.5$, the NA62 constraint is shown in green and a hypothetical future measurement of $R^0_{\nu \bar{\nu }}=10\pm 1$ is shown in orange. When $\Delta _{L}^{sd}(Z^{\prime })=0$ there is no NP contribution and this therefore corresponds to the SM

Regarding points 2 and 3, we have already seen in the analysis in Sect. 2.1.2 that for particular values of the phase $$\beta _X$$ it is possible in the presence of a SM-like value for the $${K^+ \rightarrow \pi ^+\nu \bar{\nu }}$$ branching ratio to enhance the $${K_L \rightarrow \pi ^0\nu \bar{\nu }}$$ branching ratio by an order of magnitude. Within this explicit $Z^{\prime }$ scenario, the allowed phase-space depends on the product of $$\Delta _{L}^{\ell \bar{\ell }}(Z^{\prime })\Delta _{L}^{sd}(Z^{\prime })$$. The NA62-allowed region for $$\Delta _L^{sd}(Z')$$ is an annulus in the plane of $$\left( \text {Re}\left( \Delta _{L}^{sd}(Z^{\prime })\right) , \text {Im}\left( \Delta _{L}^{sd}(Z^{\prime })\right) \right) $$, whose size depends on the chosen value of $$\Delta _{L}^{\ell \bar{\ell }}$$, as shown in the left panel of Fig. 9.

The right panel of Fig. 9 shows how a hypothetical future measurement of $R_{\nu \bar{\nu }}^{0} = 10\pm 1$ by the KOTO II experiment would significantly constrain the allowed coupling parameters, with a residual four-fold degeneracy as discussed in Sect. 2.1.2. In what follows, we will assume $R_{\nu \bar{\nu }}^{+}=1$ and set $\Delta _L^{\ell \bar{\ell }}(Z') = 0.5$, leaving us with an allowed set of values for $\Delta _{L}^{sd}(Z^{\prime })$ which define a circle in the $\left( \text {Re}\left( \Delta _{L}^{sd}(Z^{\prime })\right) , \text {Im}\left( \Delta _{L}^{sd}(Z^{\prime })\right) \right)$ plane.

Next on our list is the constraint on $\mathcal {B}(K_{L}\rightarrow \mu ^{+}\mu ^{-})_\textrm{SD}$ from (36) which implies $R_{\mu ^{+}\mu ^{-}}^{L} < 3$, see point 4. $R_{\mu ^{+}\mu ^{-}}^{L}$ depends on the product $(\Delta _{R}^{\ell \bar{\ell }}(Z') - \Delta _{L}^{\ell \bar{\ell }}(Z')) \cdot \text {Re}\left( \Delta _{L}^{sd}(Z')\right)$. Therefore, the requirement $R_{\mu ^{+}\mu ^{-}}^{L} < 3$ restricts the allowed parameter space in Fig. 9 to a vertical band centred around $\text {Re}\left( \Delta _{L}^{sd}(Z')\right) =0$, whose width depends on the value of $(\Delta _{R}^{\ell \bar{\ell }}(Z') - \Delta _{L}^{\ell \bar{\ell }}(Z'))$. In some coupling scenarios this constraint would therefore exclude two of the four possible solutions shown in the right panel of Fig. 9.

All remaining observables on our list, namely $R_{\mu ^+\mu ^-}^S$ (point 5), $R_{\pi \ell ^{+}\ell ^{-}}^{0}$ (point 6), and $\kappa _{\varepsilon ^{\prime }}$ (point 7) are sensitive to CP-violating NP contributions and therefore depend on $\text {Im}\left( \Delta _{L}^{sd}(Z')\right)$. Yet they differ in their dependence on the flavour-conserving couplings $\Delta _{L,R}^{\ell \bar{\ell }}(Z')$ and $\Delta _R^{q\bar{q}}(Z')$. The experimental constraints on $\mathcal{B}(K_L\rightarrow \pi ^0\ell ^+\ell ^-)$ from the KTeV experiment (see (54)), combined with the SM predictions (see (55) and (56)), correspond to constraints of

82 $$\begin{aligned}  &   R_{\pi e^{+}e^{-}}^{0}< 8 \, (18) \nonumber \\  &   \qquad R_{\pi \mu ^{+}\mu ^{-}}^{0} < 27 \, (40)\,\,, \end{aligned}$$

if there is positive (negative) interference between the direct and mixing-induced contributions.^6^

**Fig. 10:**
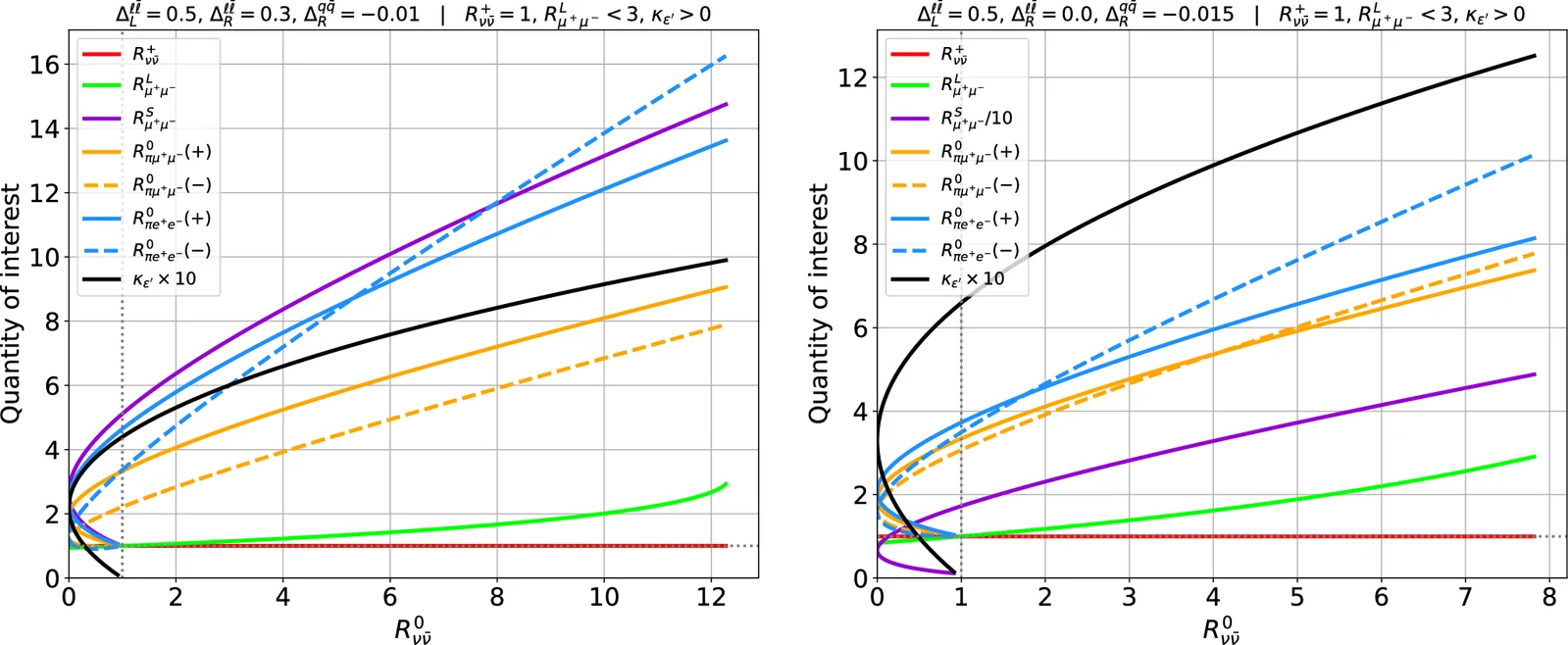
$$R_{\nu \bar{\nu }}^+$$, $$R_{\mu ^+\mu ^-}^{L,S}$$, $$R^{0}_{\pi \ell ^{+}\ell ^{-}}$$ and $$\kappa _{\varepsilon '}$$ as functions of $$R_{\nu \bar{\nu }}^0$$ for two different choices of the flavour-conserving $Z^{\prime }$ coupling parameters, enforcing $$R_{\nu \bar{\nu }}^+ = 1$$, $$R_{\mu ^+\mu ^-}^L<3$$ and $$\kappa _{\varepsilon '} > 0$$. For $$R^{0}_{\pi \ell ^{+}\ell ^{-}}$$, the solid (dashed) lines show the case of positive (negative) interference between direct and mixing-induced contributions. Left: $$\Delta _{L}^{\ell \bar{\ell }}(Z^{\prime }) = 0.5$$, $$\Delta _{R}^{\ell \bar{\ell }}(Z^{\prime }) = 0.3$$, $$\Delta _R^{q\bar{q}}(Z') = -0.01$$. Right: $$\Delta _{L}^{\ell \bar{\ell }}(Z^{\prime }) = 0.5$$, $$\Delta _{R}^{\ell \bar{\ell }}(Z^{\prime }) = 0$$, $$\Delta _R^{q\bar{q}} (Z')= -0.015$$

**Fig. 11:**
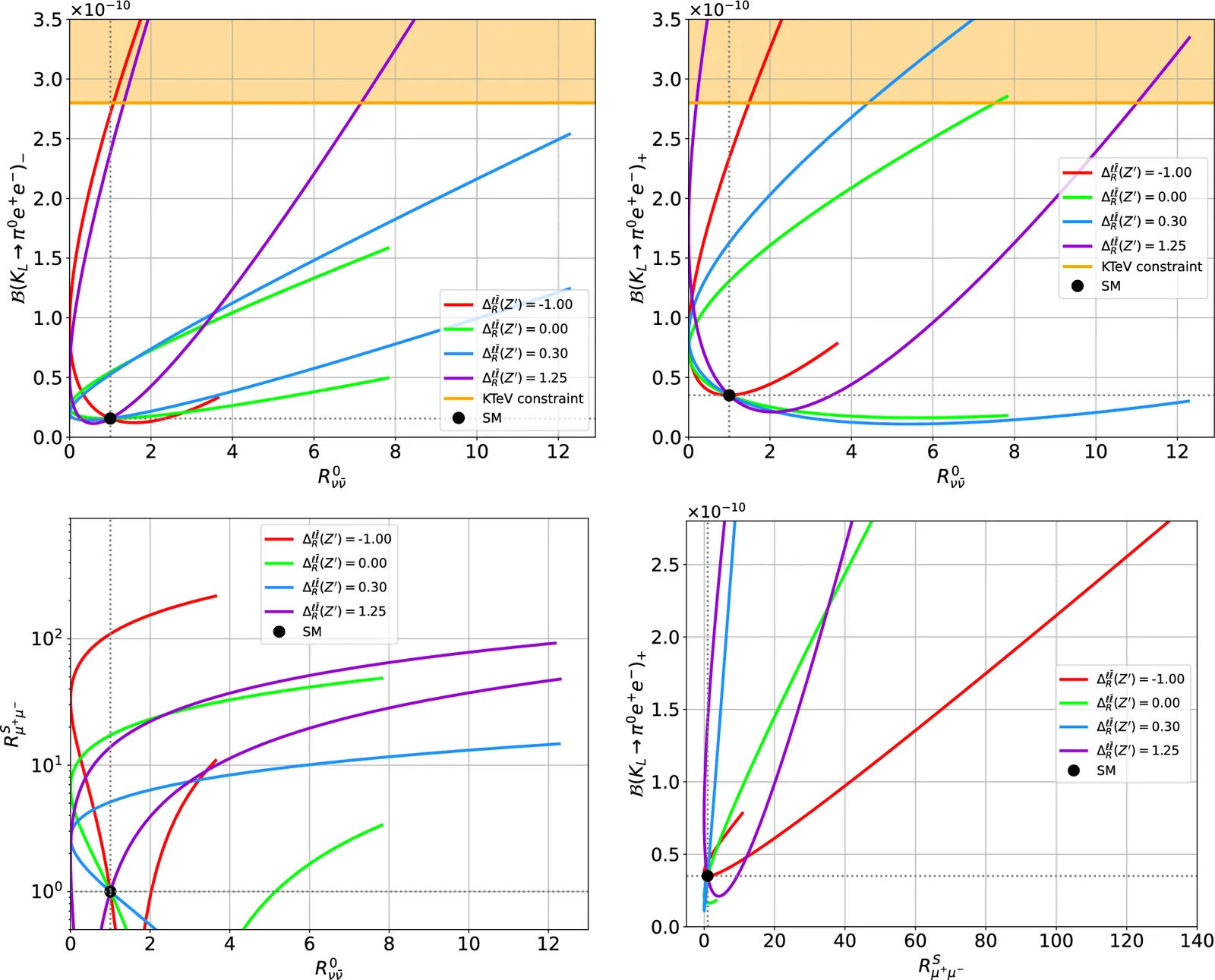
Top: comparison of $\mathcal {B}(K_{L}\rightarrow \pi ^{0}e^{+}e^{-})$ versus $R_{\nu \bar{\nu }}^{0}$ when assuming negative (left) and positive (right) interference. The experimental constraint on $\mathcal {B}(K_{L}\rightarrow \pi ^{0}e^{+}e^{-})<2.8\times 10^{-11}$ from the KTeV experiment [13] (see Eq. (54)) is shown in orange, where the shaded region is excluded at 90% C.L. Lower left: study of $R_{\mu ^{+}\mu ^{-}}^{S}$ versus $R_{\nu \bar{\nu }}^{0}$. Lower right: Correlation between the $R_{\mu ^{+}\mu ^{-}}^{S}$ variable and the branching ratio $\mathcal {B}(K_{L}\rightarrow \pi ^{0}e^{+}e^{-})$ (assuming positive interference). In all cases $\Delta _{L}^{\ell \bar{\ell }}(Z^{\prime })=0.5$ is used, the constraints $R_{\nu \bar{\nu }}^{+}=1$ and $R_{\mu ^{+}\mu ^{-}}^{L}<3$ are imposed, and results using several values of $\Delta _{R}^{\ell \bar{\ell }}(Z^{\prime })$ are shown

In Fig. 10 we show the quantities of interest (see (81)) $$R_{\mu ^+\mu ^-}^{L,S}$$, $$R_{\pi \ell ^{+}\ell ^{-}}^{0}$$ and $$\kappa _{\varepsilon '}$$ as functions of $$R_{\nu \bar{\nu }}^0$$ for two different choices of the flavour-conserving $Z^{\prime }$ couplings ($$\Delta _{L}^{\ell \bar{\ell }}(Z^{\prime })$$, $$\Delta _{R}^{\ell \bar{\ell }}(Z^{\prime })$$ and $$\Delta _{L}^{q\bar{q}}(R^{\prime })$$), as indicated in the caption. As discussed above, we enforce $$R_{\nu \bar{\nu }}^+ = 1$$—shown in red—which determines the shape of the curves. The extension of the curves to large $$R_{\nu \bar{\nu }}^0$$ values in the right panel is limited by the $$R_{\mu ^+\mu ^-}^{L}$$ bound. Finally, the requirement $$\kappa _{\varepsilon '} \ge 0$$ implies $$\text {Im}\left( \Delta _{L}^{sd}(Z')\right) \ge 0$$ for our choice of negative $$\Delta _R^{q\bar{q}}(Z')$$. This determines the other endpoints of the curves.

We observe that in both cases all requirements (points 1–7) from our list are fulfilled simultaneously. In particular, in the scenario shown in the left panel of Fig. 10, the Grossman-Nir bound for ${K_L \rightarrow \pi ^0\nu \bar{\nu }}$ can be reached and simultaneously all other observables can be significantly enhanced. When assuming positive interference between the direct and mixing-induced contributions to $K_L\rightarrow \pi ^0e^+e^-$ we find $R_{\pi e^{+}e^{-}}^{0}(+)>8$ for $R_{\nu \bar{\nu }}^0{\mathop {_\sim }\limits ^{>}}4.5$, which is excluded experimentally according to (82). In the negative interference scenario the ratio $R_{\pi e^{+}e^{-}}^{0}(-)$ remains below the experimental constraint for the full range of allowed $R_{\nu \bar{\nu }}^{0}$. (See also the $\Delta _{R}^{\ell \bar{\ell }}(Z^{\prime })=0.3$ curves in the top panel of Fig. 11 below.) Yet, even in the negative-interference case much larger decay rates than predicted in the SM are expected.

Specifically, for the coupling parameters shown in the left panel of Fig. 10, when $$R_{\nu \bar{\nu }}^0$$ reaches the Grossman-Nir bound while $$R_{\nu \bar{\nu }}^+=1$$, the bound $$R_{\mu ^+\mu ^-}^L <3$$ is saturated. At the same time, the branching ratios $$\mathcal{B}(K_L\rightarrow \pi ^0e^+e^-) = 25\times 10^{-11}$$ and $$\mathcal{B}(K_L\rightarrow \pi ^0\mu ^+\mu ^-) = 7.5 \times 10^{-11}$$ are predicted for the required negative interference. The ratio $$R_{\mu ^+\mu ^-}^S$$ reaches values as high as 15 while $$\kappa _{\varepsilon '}$$ can be $$\mathcal {O}(1)$$. Note that in the future, once the required amount of NP in $$\varepsilon '/\varepsilon $$ is more precisely known, the size of the $Z^{\prime }$ contribution can be changed by adjusting the value of $$\Delta _R^{q\bar{q}}(Z')$$ accordingly, without affecting any of the other results presented here.

Comparing the two coupling scenarios shown in Fig. 10, we identify some differences in the quantitative pattern of effects. The vanishing $Z^{\prime }$ coupling to RH leptons in the right panel leads to a significantly larger axial-vector coupling $$\Delta _A^{\ell \bar{\ell }}(Z')$$ entering the $$K_{L,S}\rightarrow \mu ^+\mu ^-$$ decays. The bound on $$R_{\mu ^+\mu ^-}^L$$ therefore does not leave enough room for $$\text {Re}\left( \Delta _{L}^{sd}(Z')\right) $$ to let $${K_L \rightarrow \pi ^0\nu \bar{\nu }}$$ reach the Grossman-Nir bound. However, large enhancements of that branching ratio, up to almost a factor of 8, are still possible. At the same time, both positive and negative interference patterns remain allowed for $$K_L\rightarrow \pi ^0\ell ^+\ell ^-$$ and both branching ratios can be significantly enhanced. We also notice that $$R_{\mu ^+\mu ^-}^S$$ can grow much larger in the right panel which is again due to the larger axial-vector coupling $$\Delta _A^{\ell \bar{\ell }}(Z')$$. Finally the larger value of $$|\Delta _R^{q\bar{q}}(Z')|$$ implies again possible $$\mathcal {O}(1)$$ values of $$\kappa _{\epsilon '}$$ despite the smaller $$\text {Im}\left( \Delta _L^{sd}(Z')\right) $$, as expected from the linear dependence of $$\kappa _{\varepsilon ^{\prime }}$$ on the $$\Delta _R^{q\bar{q}}(Z')$$ coupling parameter.

A more detailed investigation of the possible enhancements of the rates of the $K_{S}\rightarrow \mu ^{+}\mu ^{-}$ and $K_{L}\rightarrow \pi ^{0}e^{+}e^{-}$ decays, of interest for future study at the LHCb and KOTO II experiments, respectively, are shown in Fig. 11. Keeping $\Delta _{L}^{\ell \bar{\ell }}(Z^{\prime })=0.5$, and imposing the constraints $R_{\nu \bar{\nu }}^{+}=1$ and $R_{\mu ^{+}\mu ^{-}}^{L}<3$, various values of $\Delta _{R}^{\ell \bar{\ell }}(Z^{\prime })$ are considered. In the top panel a comparison of $\mathcal {B}(K_{L}\rightarrow \pi ^{0}e^{+}e^{-})$ assuming positive or negative interference between direct and mixing-induced contributions (see (45)) is shown: this highlights the significant differences in the branching ratio both for different signs of the interference and different choices of $\Delta _{R}^{\ell \bar{\ell }}(Z^{\prime })$. In the lower left panel the values of $R_{\mu ^{+}\mu ^{-}}^{S}$ versus $R^{0}_{\nu \bar{\nu }}$ are shown. We find possible values of $R_{\mu ^{+}\mu ^{-}}^{S}$ of more than 100, well within the range of the future sensitivity of LHCb. The lower-right panel then shows the correlation between $\mathcal {B}(K_{L}\rightarrow \pi ^{0}e^{+}e^{-})$ (assuming positive interference) and $R_{\mu ^{+}\mu ^{-}}^{S}$. With all considered choices of $\Delta _{R}^{\ell \bar{\ell }}(Z^{\prime })$ a significant enhancement of $\mathcal {B}(K_{L}\rightarrow \pi ^{0}e^{+}e^{-})$ is possible, saturating the experimental constraint which corresponds to the maximum vertical extent of the lower-right panel. In general $K_{S}\rightarrow \mu ^{+}\mu ^{-}$ is also enhanced substantially. We find that a scenario where only $K_{S}\rightarrow \mu ^{+}\mu ^{-}$ is enhanced and $K_{L}\rightarrow \pi ^{0}e^{+}e^{-}$ is SM-like is disfavoured in this particular model, given the existing experimental constraints. However, the opposite case, $K_{S}\rightarrow \mu ^{+}\mu ^{-}$ being SM-like while $K_{L}\rightarrow \pi ^{0}e^{+}e^{-}$ is significantly enhanced, is possible. Therefore, studies of both of these decays are essential.

## Summary

Rare and CP-violating kaon decays are among the most promising targets in the search for NP at high scales. We have demonstrated in the present article that this statement remains true after the recent NA62 measurement of $\mathcal{B}({K^+ \rightarrow \pi ^+\nu \bar{\nu }})$ consistent with the SM expectation, and despite the good agreement of the $K^0-\bar{K}^0$ mixing observables with their SM predictions. While at first sight the agreement of the NA62 result for $\mathcal{B}({K^+ \rightarrow \pi ^+\nu \bar{\nu }})$ with its SM prediction may seem disappointing, it is a very important result with profound implications for the pattern of possible NP effects in other rare kaon decays.

We have first investigated the impact of NP contributions on the $K\rightarrow \pi \nu \bar{\nu }$ decays in a model-independent analysis, stressing that in order to achieve values of $\mathcal{B}({K_L \rightarrow \pi ^0\nu \bar{\nu }})$ near the Grossman-Nir bound, a large phase $\beta _X \approx 110^\circ$ of the relevant function $X_\text {eff}$ is required. We have also demonstrated how measurements of the ${K^+ \rightarrow \pi ^+\nu \bar{\nu }}$ and ${K_L \rightarrow \pi ^0\nu \bar{\nu }}$ branching ratios by NA62 and KOTO II, respectively, can be used to determine the values of $|X_\text {eff}|$ and $\beta _X$.

We have then turned our attention to a specific $Z^{\prime }$ scenario with flavour-violating *sd* couplings, in order to correlate the possible NP pattern in $$K\rightarrow \pi \nu \bar{\nu }$$ with other kaon decays of interest, in particular the neutral kaon mixing observables $$\varepsilon _K$$ and $$\Delta M_K$$, the branching ratios of the decays $$K_{L,S}\rightarrow \mu ^+\mu ^-$$ and $$K_L\rightarrow \pi ^0\ell ^+\ell ^-$$, as well as the parameter $$\varepsilon '/\varepsilon $$ measuring direct CP violation in $$K\rightarrow \pi \pi $$. Our analysis proves that large enhancements of the decays $${K_L \rightarrow \pi ^0\nu \bar{\nu }}$$, $$K_L\rightarrow \pi ^0\ell ^+\ell ^-$$ and $$K_S\rightarrow \mu ^+\mu ^-$$ as well as of the parameter $$\varepsilon '/\varepsilon $$ remain simultaneously possible despite the $$K^0-\bar{K}^0$$ observables being in good agreement with the SM prediction and the constraint from $$K_L\rightarrow \mu ^+\mu ^-$$, even in the most pessimistic scenario of no NP in the decay $${K^+ \rightarrow \pi ^+\nu \bar{\nu }}$$.

It should be emphasised that the $Z^\prime$ scenario considered by us is only an illustration of a particular NP scenario at work, and other more complete NP models may predict somewhat different patterns of effects.

Our work thus strongly encourages the continuation of the experimental kaon physics programme, with future measurements of the decays ${K_L \rightarrow \pi ^0\nu \bar{\nu }}$, $K_L \rightarrow \pi ^0e^+e^-$ and possibly $K_L\rightarrow \pi ^0\mu ^+\mu ^-$ by the KOTO II experiment as well as of $K_S\rightarrow \mu ^+\mu ^-$ by LHCb, to be complemented by continued efforts by the theory and lattice communities to improve the SM prediction of $\varepsilon '/\varepsilon$. On the theory side, the NA62 result already has an impact on the correlations between rare kaon and *B* meson decays in concrete NP scenarios, as evident from the DNA-charts in [87]. Indeed, the first study of the implications of the NA62 result on such correlations appeared recently [88]. Yet, the full assessment will only be possible when the data on rare *B* and *K* decays improves in the coming years.

## Data Availability

This manuscript has no associated data. [Authors’ comment: Data sharing not applicable to this article as no datasets were generated or analysed during the current study.]

## A CKM Parameters from BV Strategy

Basing the determination of CKM parameters exclusively on ΔF=2 observables as done in [16] and updating the value of $\beta =22.62(45)^\circ$ from HFLAV [89] relative to β=22.2(7) used in [16] one finds [25]

83 $$\begin{aligned}  &   |V_{cb}|=42.5(5)\times 10^{-3}, \quad \gamma =64.6(16)^\circ , \quad \nonumber \\  &   \beta =22.62(45)^\circ , \quad |V_{ub}|=3.76(11)\times 10^{-3}\, \end{aligned}$$

and consequently

84 $$\begin{aligned}  &   |V_{ts}|=41.8(4)\times 10^{-3}, \qquad \nonumber \\  &   \beta _{s} = -1.05(4)^{\circ }, \qquad {|V_{td}|=8.65(15)\times 10^{-3}}. \end{aligned}$$

We then have

85 $$\begin{aligned}  &   {\mathrm{{Re}}\lambda _c = -0.2189(9),\quad \mathrm{{Re}}\lambda _t = -3.31(10) \times 10^{-4},\quad } \nonumber \\  &   {{\text {Im}}\lambda _t=1.44(5)\times 10^{-4},} \end{aligned}$$

where $$\lambda _c=V_{cs}^*V_{cd}$$ and $$\lambda _t=V_{ts}^*V_{td}$$.

## Footnotes

1 The impressive progress made by the NA62 collaboration on ${K^+ \rightarrow \pi ^+\nu \bar{\nu }}$ and by KOTO on ${K_L \rightarrow \pi ^0\nu \bar{\nu }}$ becomes evident when reading the extensive review on the status and perspectives on both decays written in 2015 just after the start of the NA62 experiment [5].

2 Note however the strong dependence of $\varepsilon _{K}$ on the CKM parameter $|V_{cb}|$, calling for a solution of the tensions in the determination of the latter parameter.

3 In this paper, we restrict our attention to the case of SM neutrinos in the final state.

4 Note that constraints from *B* meson decays can only be meaningfully incorporated once a NP flavour structure is assumed. For instance [50] assumed a new flavour-universal contribution to the SM $Z^0$-penguin loop function $C(x_t)$.

5 Note that now $\bar{\beta }_Y$ and $\bar{\theta }_Y$ have to be introduced as the $K_{L}\rightarrow \pi ^{0}\ell ^{+}\ell ^{-}$ decays are sensitive to the $\bar{s}d$ vector current.

6 Both the numerator and denominator entering $R_{\pi \ell ^{+}\ell ^{-}}^{0}$ change between the positive and negative interference scenarios.

## Code Availability Statement

This manuscript has no associated code/software. [Authors’ comment: Code/software sharing not applicable to this article as no code/software was generated or analysed during the current study.]
